# Pharmacological chaperone action in humanized mouse models of MC4R-linked obesity

**DOI:** 10.1172/jci.insight.132778

**Published:** 2021-02-22

**Authors:** Patricia René, Damien Lanfray, Denis Richard, Michel Bouvier

**Affiliations:** 1 Départment de Biochimie et de Médecine Moléculaire, Institute for Research in Immunology and Cancer, Université de Montréal, Montréal, Quebec, Canada.; 2 CRIUCPQ, Université Laval, Québec City, Québec, Canada.

**Keywords:** Metabolism, Therapeutics, G protein&ndash;coupled receptors, Melanocortin, Obesity

## Abstract

MC4R mutations represent the largest monogenic cause of obesity, resulting mainly from receptor misfolding and intracellular retention by the cellular quality control system. The present study aimed at determining whether pharmacological chaperones (PCs) that restore folding and plasma membrane trafficking by stabilizing near native protein conformation may represent valid therapeutic avenues for the treatment of melanocortin type 4 receptor–linked (MC4R-linked) obesity. To test the therapeutic PC potential, we engineered humanized MC4R (hMC4R) mouse models expressing either the WT human MC4R or a prevalent obesity-causing mutant (R165W). Administration of a PC able to rescue cell surface expression and functional activity of R165W-hMC4R in cells restored the anorexigenic response of the R165W-hMC4R obese mice to melanocortin agonist, providing a proof of principle for the therapeutic potential of MC4R-targeting PCs in vivo. Interestingly, the expression of the WT-hMC4R in mice revealed lower sensitivity of the human receptor to α–melanocyte-stimulating hormone (α-MSH) but not β-MSH or melanotan II, resulting in a lower penetrance obese phenotype in the WT-hMC4R versus R165W-hMC4R mice. In conclusion, we created 2 new obesity models, a hypomorphic highlighting species differences and an amorphic providing a preclinical model to test the therapeutic potential of PCs to treat MC4R-linked obesity.

## Introduction

Severe obesity is generally considered a multifactorial disease caused by both genetic and environmental factors resulting in an energy imbalance. The predisposition to obesity is largely polygenic but can also be monogenic (1, 2). Among these, mutations occurring in the melanocortin type 4 receptor (MC4R), a key component of the central regulation of energy homeostasis, have been associated with severe familial obesity and reported as the most common monogenic cause of childhood obesity (3–6). The prevalence of MC4R mutations in obese people varies substantially in frequency (1%–6% of the obese patients) depending on the ethnic origin, the severity of obesity, and the age of obesity onset of the population considered (7). Although some subjects are homozygous for MC4R mutations, the vast majority are heterozygous. The mode of inheritance is defined as autosomal codominant with modulation of expression and of phenotype penetrance (6).

More than 150 distinct mutations causing early-onset obesity have so far been identified, and, from those characterized, a majority causes protein misfolding leading to the retention of the receptor by the cell quality control system, thus preventing its normal trafficking to the cell surface (8). These defects belong to a growing class of so-called conformational mutations that affect members of different protein families, including many GPCRs (9). Several years ago, cell-permeant, pharmacologically selective ligands that bind misfolded proteins and promote their proper folding were identified and called pharmacological chaperones (PCs), (9, 10). Such compounds favor the escape of mutant proteins that are otherwise retained in the endoplasmic reticulum or Golgi by the quality control system of the cell as a consequence of their improper folding. By penetrating the cell membrane and binding to the misfolded receptor, these PCs stabilize a near native conformation, allowing their trafficking to the plasma membrane. In many cases, such rescued receptors maintained their capacity to engage their cognate signaling pathway, thus restoring function. PCs therefore represent an attractive therapeutic avenue for the treatment of “conformational diseases.” In most cases, PCs bind the orthosteric binding pocket of the targeted receptor. Compounds with antagonist activity are generally preferred over agonists because they do not promote desensitization or downregulation. This implies that to be therapeutically active, the pharmacokinetic properties of the PCs must allow their dissociation from the receptor, allowing binding of the natural agonist. The therapeutic efficacy of such a PC approach on GPCRs was demonstrated in vivo in a hypogonadotropic hypogonadal mouse model harboring a misfolded gonadotropin-releasing hormone receptor (GnRHR) (11) and in a pilot clinical trial for the treatment of nephrogenic diabetes insipidus targeting mutant forms of vasopressin type 2 receptor (12). Development of therapeutic strategies targeting rare forms of early-onset obesity would represent a significant advance in pediatric obesity, especially when considering that current dietary approaches and pharmacotherapy are largely ineffective and that bariatric surgery is a rather invasive intervention that is not recommended in children or teenagers (13–17). Our previous study in cultured cells demonstrated the efficacy of membrane-permeant MC4R-selective PCs to restore cell surface expression and function of MC4R mutant forms, paving the way toward the development of a therapy targeting patients with MC4R-related obesity (18).

The only mouse models currently available and mimicking MC4R deficiency are MC4R-knockout models that do not allow the assessment of therapeutic candidates acting as PCs, which require models producing misfolded receptors that are retained intracellularly. To address the therapeutic value of PCs in vivo to treat MC4R-linked obesity, we generated humanized MC4R knockin mouse lines expressing either the WT or an obesity-causing mutant form of the human MC4R (hMC4R) in the receptor’s mouse locus. We inserted the naturally occurring mutation Arg165Trp (R165W), which was previously described as retained intracellularly in heterologous expression systems (8) and was found to be one of the prevalent mutations (number of probands) found in severely obese patients (19–23). This mutant form of the receptor was chosen since we previously found that PCs could efficiently rescue both its cell surface expression and function in cellular systems (18).

Here, we report the development of 2 mechanistically distinct MC4R-linked obesity models. Mice carrying the WT allele of the human MC4R (WT-hMC4R) in place of the mouse MC4R (mMC4R) gene developed a “hypomorphic” phenotype leading to obesity because of a lower sensitivity of the human receptor to α–melanocyte-stimulating hormone (α-MSH) as compared with the mouse receptor. In contrast, the obesity observed in mice carrying the mutant R165W-hMC4R allele in place of the mMC4R gene was due to the insertion of an amorphic allele, the presence of R165W mutation leading to the expression of a nonfunctional receptor in vivo. We used this model, mimicking features of human conformational MC4R obesity, to assess in vivo the potential efficacy of our MC4R-selective PC candidate, PC UM0130866. Oral (per os) administration of this PC promoted the restoration of the anorexic effect of the MC4R agonist, melanotan II (MTII), in mice harboring the obesity-causing mutant form R165W-hMC4R but not in the *loxP*-flanked transcriptional blocking (loxTB) MC4R-null mice. In addition to providing 2 mechanistically distinct mouse models for MC4R-linked obesity, the study presents the first in vivo demonstration to our knowledge of MC4R-specific PC action, paving the way for the development of a targeted therapy for severely obese patients harboring MC4R mutations.

## Results

### Generation and validation of hMC4R knockin mouse models.

To explore the potential of a PC therapeutic approach in the context of MC4R deficiency in humans, we generated knockin (KI) mouse lines expressing the WT or an obesity-causing mutant form of the hMC4R in the receptor’s mouse locus. We used homologous recombination in embryonic stem (ES) cells to replace the mouse *mc4r* coding gene by either the human WT MC4R tagged at the N-terminus with a c-myc epitope or a mutant form of the human MC4R carrying an obesity-linked mutation at position R165**→**W in the hMC4R coding sequence tagged at the N-terminus with 3 tandem sequences of the HA epitope. To facilitate the detection of the transgene expression, both forms of the human MC4R were fused in frame at the C-terminus with the Venus fluorescent protein flanked by *loxP* sites. A detailed description of the knockin strategy is presented in Supplemental Figure 1A; supplemental material available online with this article; https://doi.org/10.1172/jci.insight.132778DS1; and Supplemental Methods. Homologous recombination at the receptor mouse locus was confirmed by Southern blot analysis (Supplemental Figure 1, A and B) and validated by PCR through the genotyping of the mice.

**Figure 1:**
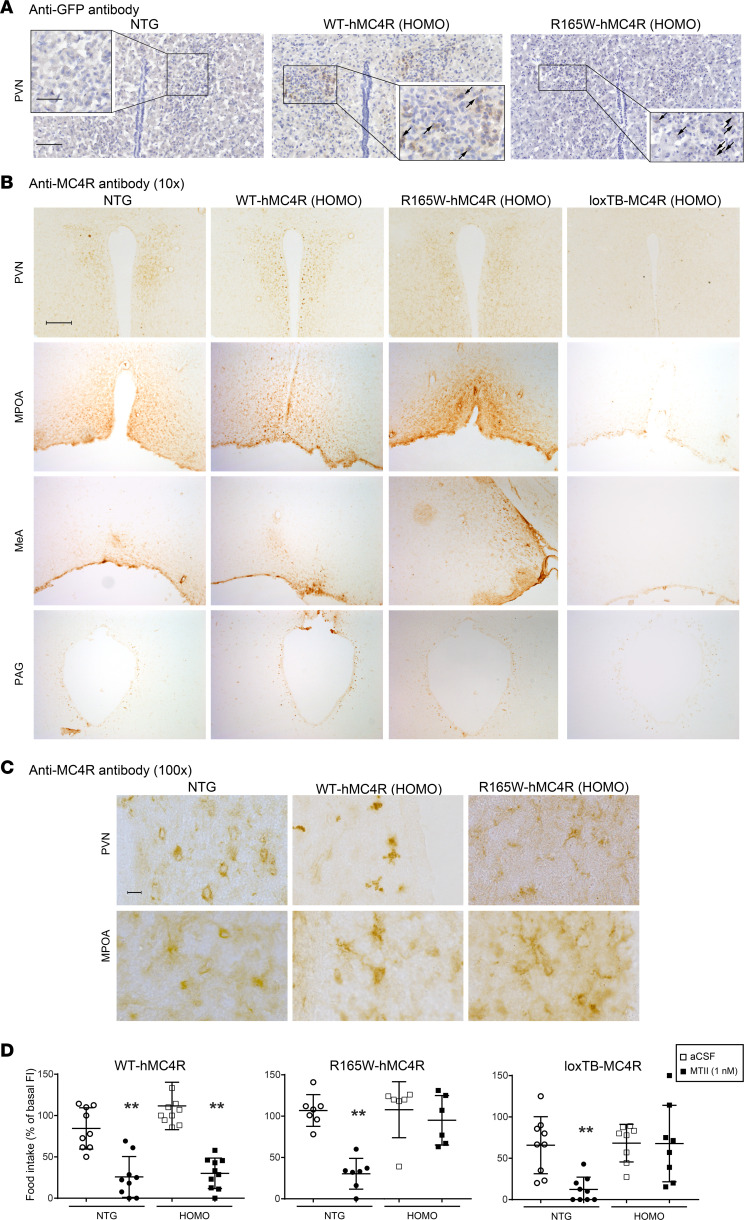
Humanized KI mouse expression and functionality. (**A**–**C**) Immunohistochemical labeling of MC4R-positive neurons. Brain sections of the paraventricular nucleus (PVN) of the hypothalamus, the medial preoptic area (MPOA), the medial amygdala (MeA), or the mesencephalic periaqueductal gray (PAG) from nontransgenic (NTG), heterozygous (HET), or homozygous (HOMO) hMC4R-KI and homozygous loxTB MC4R-null mice (as indicated) were labeled with anti-GFP antibody (**A**) or with anti-MC4R antibody (**B** and **C**), **C** being a higher magnification of the PVN and MPOA for the NTG, WT and R165W-hMC4R mice. Inset in **A**: higher power magnification of boxed areas containing GFP immune reactive neurons (brown cytoplasmic reaction product). Arrows in the center and right insets indicate GFP-positive neurons. Scale bars: 100 μm (**A**), 50 μm (**A**, inset), 2 cm (**B**) and 10 μm (**C**). (**D**) MTII response in mouse models. MTII (1 nmol) was intracerebroventricularly (icv) injected in 16- to 22-week-old nontransgenic (NTG) and homozygous (HOMO) hMC4R-KI (WT or mutant [R165W]) or loxTB MC4R-null mice. Food intake was measured 4 hours postadministration in dark phase and reported in percentage. Data were analyzed using the Wilcoxon’s matched pairs test for loxTB MC4R null (NTG = 9, *P* = 0.0078, HOMO = 10), WT-hMC4R (NTG = 9, *P* = 0.0078; HOMO = 10, *P* = 0.0020), and R165W-hMC4R (NTG = 7, *P* = 0.0156; HOMO = 6). **P < 0.001.

Immunohistochemistry on frozen brain sections in heterozygous WT-hMC4R and R165W-hMC4R mice using an anti-GFP antibody revealed Venus expression level in paraventricular nuclei of the hypothalamus known to endogenously express the mMC4R (Figure 1A). We observed a more intense GFP immunoreactivity for WT-hMC4R–expressing neurons compared with R165W-hMC4R–expressing neurons. No labeling with the GFP antibody was detected in nontransgenic littermates (Figure 1A).

To compare the labeling profile of the transgenic and the endogenous MC4R expression, we also tested a specific anti-MC4R antibody directed against an epitope conserved in human and mouse MC4R sequence located in the extracellular N-terminus (24). As shown in Figure 1, B and C, we observed the same labeling pattern in homozygous mice carrying either the WT- or the mutant-hMC4R allele as the one observed in nontransgenic mice. Immunolabelings are illustrated for the PVN, the ventromedial hypothalamus MPOA, the MeA, and the mesencephalic PAG that endogenously expressed MC4R (25–27). No labeling with this anti-MC4R antibody was detected in loxTB MC4R-null mice (Figure 1B), validating its specificity against MC4R.

Moreover, we performed quantitative reverse transcription PCR on hypothalamus extracts from hMC4R-KI mice and nontransgenic littermates. As expected, we measured the same amount of MC4R mRNA in the 3 genotypes, indicating that the hMC4R transgene allele was expressed at the same level as the mouse endogenous mc4r allele (see Supplemental Table 1).

To assess the functionality of the WT and mutant forms of the human MC4R in the transgenic mice, the food intake of animals carrying these alleles was monitored during the nocturnal cycle 4 hours following the icv administration of the melanocortin agonist, MTII, in the lateral ventricle. As shown in Figure 1D (left panel), MTII injection inhibited food intake in homozygous mice carrying the WT-hMC4R allele as efficiently as in their nontransgenic littermates, demonstrating that the tagged version of the WT-hMC4R allele was functional in vivo. As expected, no significant inhibition of food intake was observed after MTII administration in either homozygous loxTB MC4R-null mice (28) (Figure 1D, right panel) or homozygous R165W-hMC4R mice (Figure 1D, middle panel), demonstrating that the anorexigenic effect of MTII was MC4R dependent and that the naturally occurring human variant R165W-hMC4R was defective when expressed in mice as previously reported in cell-based assays (18).

### Both humanized mouse models develop obesity.

Since both knockin mouse models expressed the transgenic receptor but differed regarding the allele functionality, we studied the evolution of their phenotypes in comparison with their respective nontransgenic littermates carrying the normal mouse allele.

**Figure 2:**
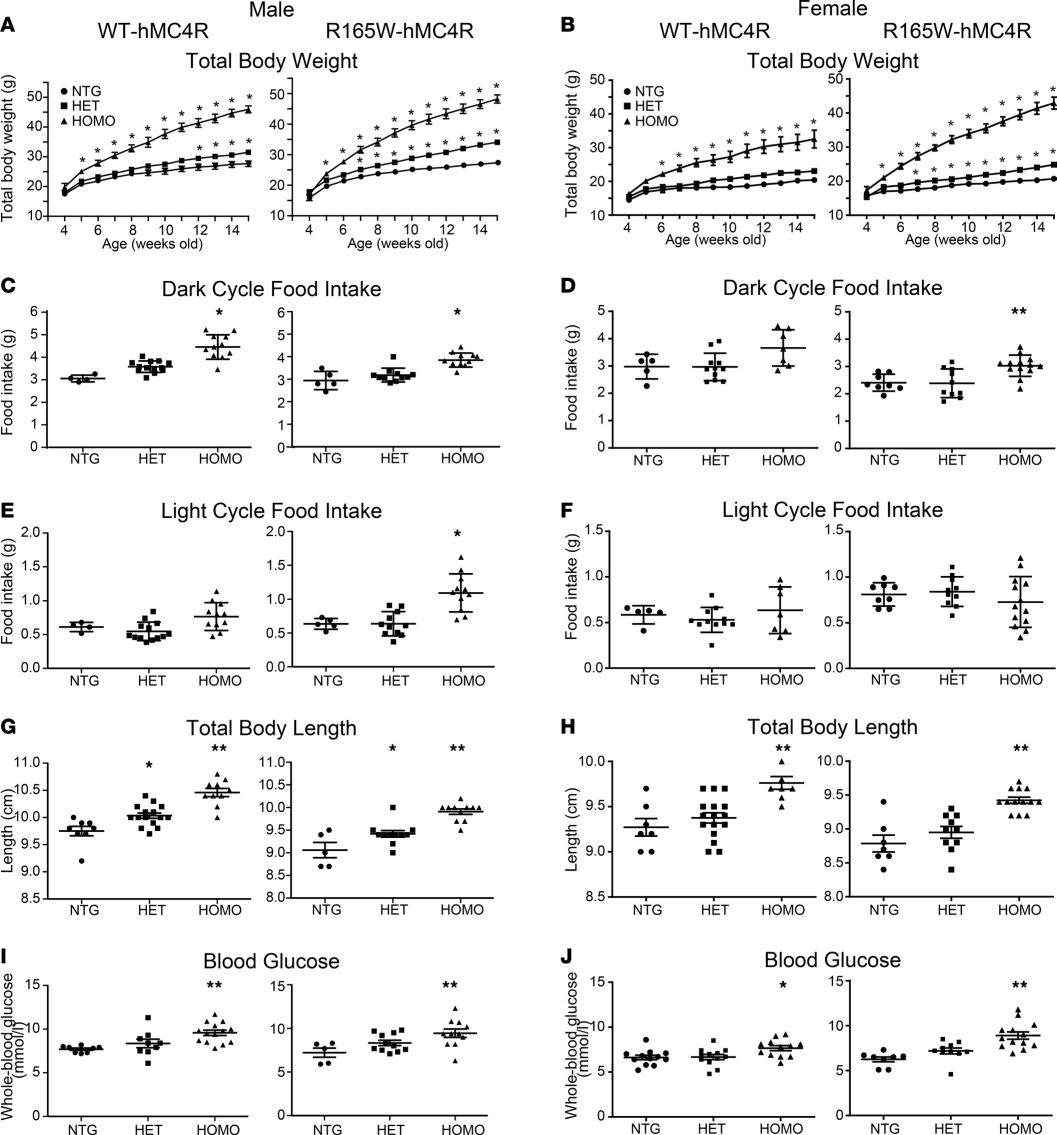
Body weight gain, food consumption, body length, and blood glucose of WT- and R165W-hMC4R-KI mice and their control littermates. (**A** and **B**) Weight gain of male (**A**) or female (**B**) WT-hMC4R-KI mice (left) (male: NTG 7, HET 15, HOMO 10; female: NTG 6, HET 14, HOMO 8) or R165W-hMC4R-KI mice (right) (male: NTG 10, HET 19, HOMO 9; female: NTG 10, HET 12, HOMO 6). (**C** and **D**) Food intake during dark cycle of WT-hMC4R (male: NTG 4; HET 13; HOMO 11; female: NTG 5, HET 11, HOMO 7) or R165W-hMC4R (male: NTG 5, HET 11, HOMO 11; female: NTG 8, HET 10, HOMO 13). (**E** and **F**) Food intake during light cycle of WT-hMC4R (male: NTG 4; HET 13, HOMO 11; female: NTG 5, HET 11, HOMO 7) or R165W-hMC4R (male: NTG 5; HET 11; HOMO 11; female: NTG 8, HET 10, HOMO 13). The caloric intake was estimated using 3.1 kcal/g as metabolizable energy. (**G** and **H**) Total body length of WT-hMC4R (male: NTG 8, HET 15, HOMO 10; female: NTG 7, HET 16, HOMO 8) or R165W-hMC4R (male: NTG 5, HET 11, HOMO 11; female: NTG 7, HET 10, HOMO 13) mice. (**I** and **J**) Blood glucose assessment in fasted mice: WT-hMC4R (male: NTG 9, HET 11, HOMO 14; female: NTG 12, HET 12, HOMO 12) or R165W-hMC4R (male: NTG 5, HET 11, HOMO 11; female: NTG 8, HET 10, HOMO 13). Data are shown as the mean ± SEM and were analyzed using 2-way ANOVA test of variance followed by pairwise comparisons using post hoc Bonferroni’s multiple comparisons test (**A** and **B**) or 1-way Kruskal-Wallis test of variance followed by pairwise comparisons using post hoc Dunn’s multiple comparisons test (**C**–**J**). Asterisks denote significant difference of either HOMO or HET hMC4R-KI mice compared with NTG littermates. *P < 0.05, **P < 0.001.

First, we monitored body weight gain weekly in offspring fed with regular chow (Figure 2, A and B). By 5 weeks of age, both male and female homozygous hMC4R mice carrying either the WT-hMC4R or the R165W-hMC4R allele were significantly heavier than their nontransgenic littermates. In heterozygous mice, the onset of increased weight gain differed between genotypes and sex. Whereas WT-hMC4R heterozygous male mice showed a significant increase in weight gain only at 12 weeks of age, heterozygous R165W-hMC4R male mice became heavier than their nontransgenic littermates as early as at 7 weeks old. The difference was even more striking in females since no significant weight gain difference was observed between heterozygous WT-hMC4R females and their littermates, even at the latest age examined, while R165W-hMC4R heterozygous females became significantly heavier than their nontransgenic siblings starting at 11 weeks old. These observations reveal that despite having a functional receptor, mice carrying the WT-hMC4R allele developed obesity on regular chow diet but differed from mice carrying the R165W-hMC4R allele both in the onset of obesity and the penetrance of the phenotype, as seen in male and female heterozygotes, respectively. These discrepancies might reflect phenotypes resulting from haploinsufficiency in the case of R165W-hMC4R heterozygous mice compared with a possible hypomorphic allele in the case of WT-hMC4R heterozygous mice.

Since MC4R plays a pivotal role in the regulation of food consumption, we next assessed whether the increased weight gain was associated with increased feeding. Measurement of food intake at 19–20 weeks during dark and light phases (Figure 2, C–F) revealed that for homozygous animals carrying either the WT-hMC4R or the R165W-hMC4R allele, significant increase in food intake compared with nontransgenic littermates was observed in the dark phase (Figure 2, C and D). However, food intake was significantly increased during the light phase only in R165W-hMC4R homozygous male mice (Figure 2E), again indicating differences between the 2 models. Consistent with a greater penetrance of the phenotype in the R165W-hMC4R carrying animals, the daily averaged food intake was found to be 57% greater than their nontransgenic littermates for the R165W-hMC4R homozygous male as compared with a 47% increase for the WT-hMC4R homozygous males. No significant change in daily food intake was observed for any of the heterozygous mice when compared with their respective littermates during the period examined. Taken together, the data reveal hyperphagia for both knockin hMC4R mouse models with a slightly more prominent effect in the R165W-hMC4R mice, consistent with a contribution of an increased food intake in the obesity phenotype, particularly in the homozygotes.

Consistent with the increased linear growth previously observed in both humans with MC4R deficiency (29) and MC4R-null mice (30), an increase in axial length was observed in male and female homozygotes and heterozygotes carrying either hWT or hR165W alleles (Figure 2, G and H). Also consistent with the phenotypes reported in MC4R-null mice, whole blood glucose was found to be significantly higher in both WT-hMC4R and R165W-hMC4R homozygous lines for the 2 sexes in fasted animals at 17 weeks old (Figure 2, I and J) (31).

Taken as a whole, these data show that the insertion of either WT- or R165W-hMC4R in the mouse MC4R locus led to 2 obesity models that recapitulated several of the characteristics observed in obese patients with MC4R deficit, albeit with a modestly lower penetrance for the WT human allele. Although this was expected for the R165W-hMC4R mice, it was unanticipated for the mice expressing the WT-hMC4R.

### WT-hMC4R homozygous mice are less sensitive to α-MSH.

**Figure 3:**
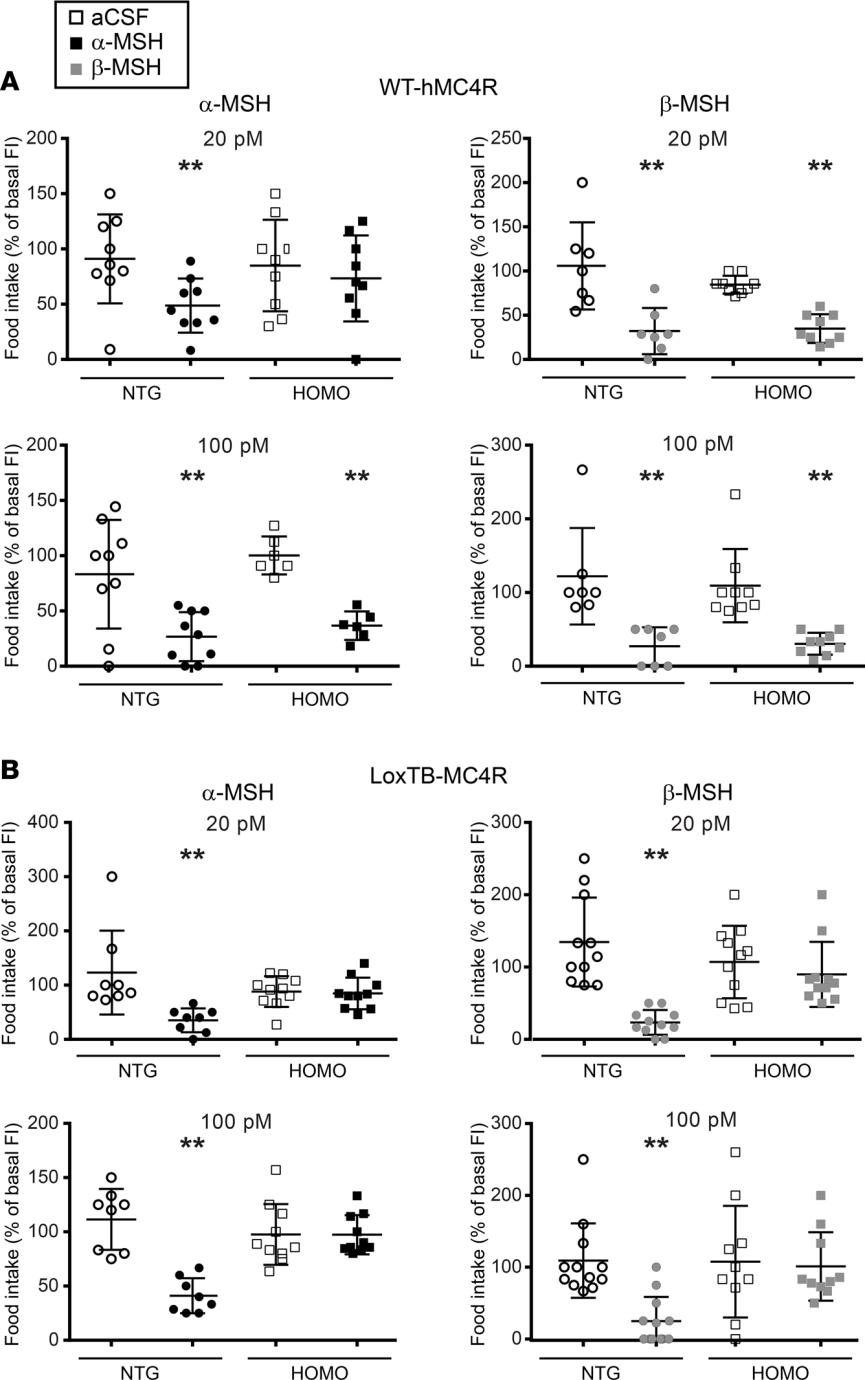
Central effect of natural melanocortin agonists on nocturnal food intake in WT-hMC4R-KI and loxTB MC4R-null mice. Icv injections in 16- to 22-week-old WT-hMC4R-KI (**A**) (NTG = 9; Homo = 10) or loxTB MC4R-null (**B**) (NTG = 9; Homo = 8) male mice. Icv injection of doses of α-MSH (left panel) or β-MSH (right panel) (20 pmol/μL and 100 pmol/μL, from upper to lower panels) were done at Zeitgeber time 10 (ZT10) (10 hours after lights on). Food consumption was then assessed between ZT10 and ZT15 (3 hours after lights off). Food consumption was then normalized relative to the basal food consumption measured the day before for each mouse (% food intake vs. basal) and is expressed as means ± SEM. The sequence of the α-MSH peptide used is shared between mouse and human whereas the human sequence was used for β-MSH since it is not produced in the mouse. The asterisks denote significant difference of α-MSH or β-MSH group compared with the group receiving artificial cerebrospinal fluid (aCSF) only, using Wilcoxon’s matched pairs signed-rank test. **P < 0.001.

Since homozygous mice carrying the WT-hMC4R allele appeared as hypomorphic despite having a functional receptor (as revealed by the normal response to MTII; see Figure 1C), we assessed the sensitivity of mice carrying the WT-hMC4R allele to the natural agonists of the receptor, the melanocortins. To directly test whether human and mouse MC4R orthologs may have different sensitivity to endogenous melanocortins, we monitored the dose-dependent effect of α- and β-MSH on food intake in homozygous WT-hMC4R mice and nontransgenic littermates (Figure 3A). The melanocortins were also administered to the loxTB MC4R-null mice used as negative controls (Figure 3B). As shown in Figure 3A, homozygous WT-hMC4R mice and their littermates had identical responses to 100 pmol/μL of α-MSH. However, the lower dose of α-MSH (20 pmol/μL) was ineffective in WT-hMC4R while still significantly inhibiting food intake in their nontransgenic littermates harboring the mouse allele. In contrast, similar anorexic responses to β-MSH (endogenously secreted in humans but not in mice due to the absence of the proper cleavage site in the mouse proopiomelanocortin; refs. 32, 33) were observed for both homozygous WT-hMC4R mice and their nontransgenic littermates at both 100 and 20 pmol/μL. The absence of anorexic response in homozygous loxTB MC4R-null mice with either ligand indicates that the effect measured in WT-hMC4R-KI mice was MC4R mediated (Figure 3B). Altogether, those results strongly suggest that, although fully responsive to MTII and β-MSH, the human receptor is less sensitive to α-MSH, pointing to a species difference in responsiveness to the endogenous melanocortins.

To test this possibility, we measured the potency of α-MSH to activate the stimulatory G alpha subunit (Gs) upon binding to either mMC4R or hMC4R using a bioluminescence resonance energy transfer–based (BRET-based) biosensor directly monitoring Gs activation (Supplemental Figure 2). We observed that the potency (the negative logarithm of the concentration leading to 50% of the maximal response, pEC_50_) of α-MSH on mMC4R to activate Gs was 14-fold higher than on hMC4R (pEC_50_: 7.48 ± 0.15 for hMC4R vs. 8.64 ± 0.22 for mMC4R). These data support the difference in the sensitivity of the human versus mouse receptor to α-MSH and may explain the development of an obese phenotype in the WT-hMC4R mice.

### Central chronic infusion of MTII reverses overweight and hyperphagia in homozygous WT-hMC4R mice.

**Figure 4:**
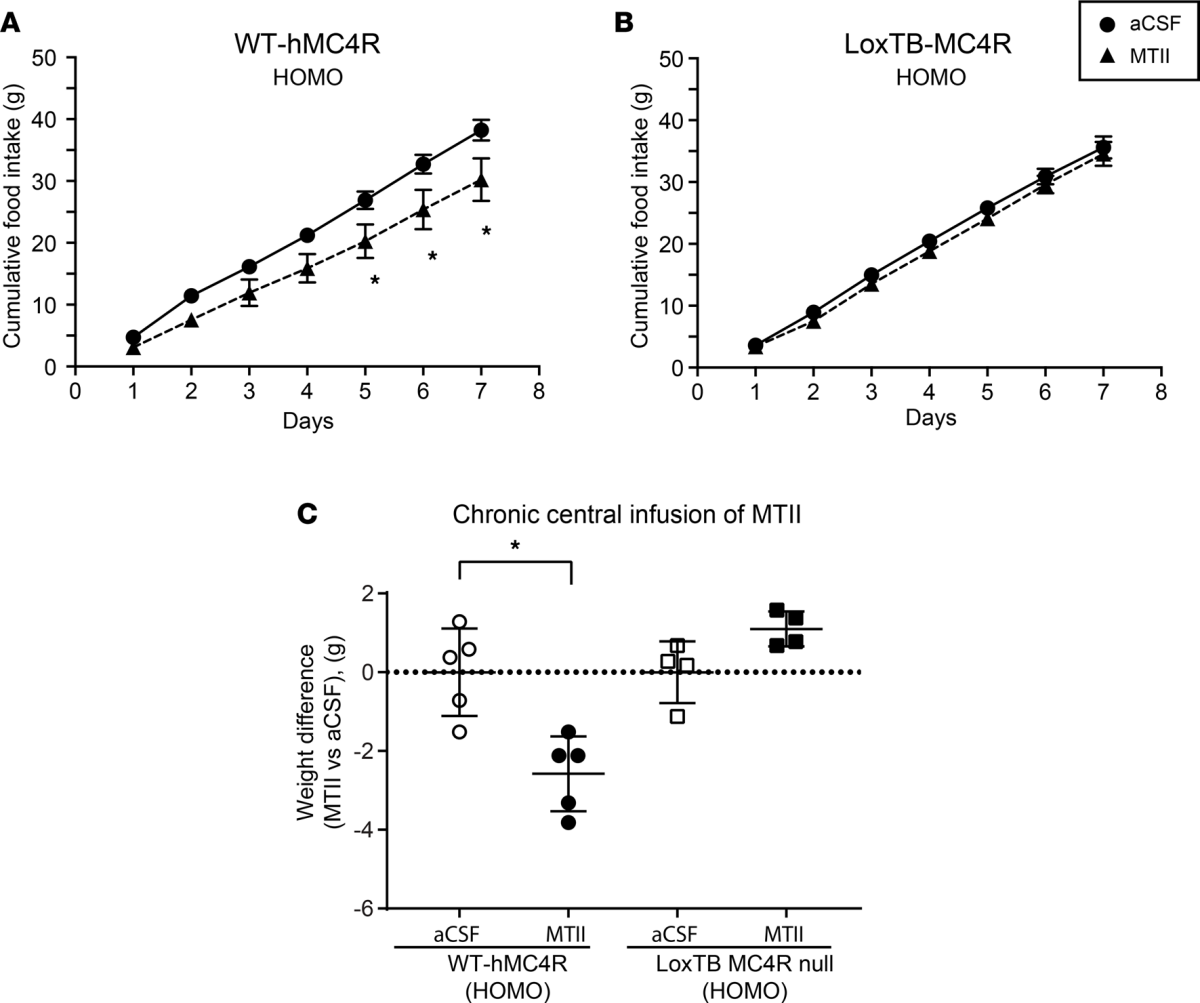
Effect of central chronic infusion of MTII on food intake and total body weight in homozygous WT-hMC4R-KI and loxTB-MC4R-null mice. MTII at a dose of 1 nM/d for 7 days was infused icv through a catheter connected to an osmotic pump implanted subcutaneously. (**A** and **B**) Food consumption was evaluated each day at ZT6 (6 hours after lights on) during 1 week. Data are the mean ± SEM of 24 hours’ food intake for WT-hMC4R male mice (**A**) (aCSF and MTII *n* = 5) or loxTB-MC4R-null male mice (**B**) (aCSF and MTII, *n* = 5). Analysis was done using 2-way ANOVA test of variance followed by pairwise comparisons using post hoc Bonferroni’s multiple comparisons test. (**C**) Body weight was evaluated each day at ZT6 (6 hours after lights on) during 1 week. Data are the mean of Δ weight loss upon MTII treatment compared with total body weight upon aCSF treatment for each group of mice for WT-hMC4R (aCSF and MTII, *n* = 5) or loxTB-MC4R (aCSF and MTII, *n* = 4). Analysis was done using the nonparametric Mann-Whitney *U* test. All experiments were done in mice at 16–20 weeks of age. The asterisks denote significant difference of HOMO hMC4R-KI or -KO mice compared with aCSF icv infusion. *P < 0.05.

To test whether the increase in food intake and weight gain in WT-hMC4R mice is due to a decrease in melanocortin agonist tone resulting from the hypomorphism of the WT human allele, we monitored the effect of chronic central administration of MTII. Upon chronic central MTII stimulation, cumulative food intake in homozygous WT-hMC4R mice was significantly less at days 5, 6, and 7 compared with animals receiving aCSF only (Figure 4A). As expected, no effect was seen in the loxTB MC4R-null mice (Figure 4B). Chronic administration of MTII also resulted in a significant body weight loss in WT-hMC4R but not in loxTB MC4R-null mice (Figure 4C). Taken together, these data suggest that the primary cause of obesity in WT-hMC4R mice was a decrease in MC4R agonist tone reflecting species differences in MC4R receptor relative to endogenous melanocortins, thus providing a potentially new model of MC4R-linked obesity.

### R165W-hMC4R homozygous mice have impaired glucose tolerance and energy expenditure.

**Figure 5:**
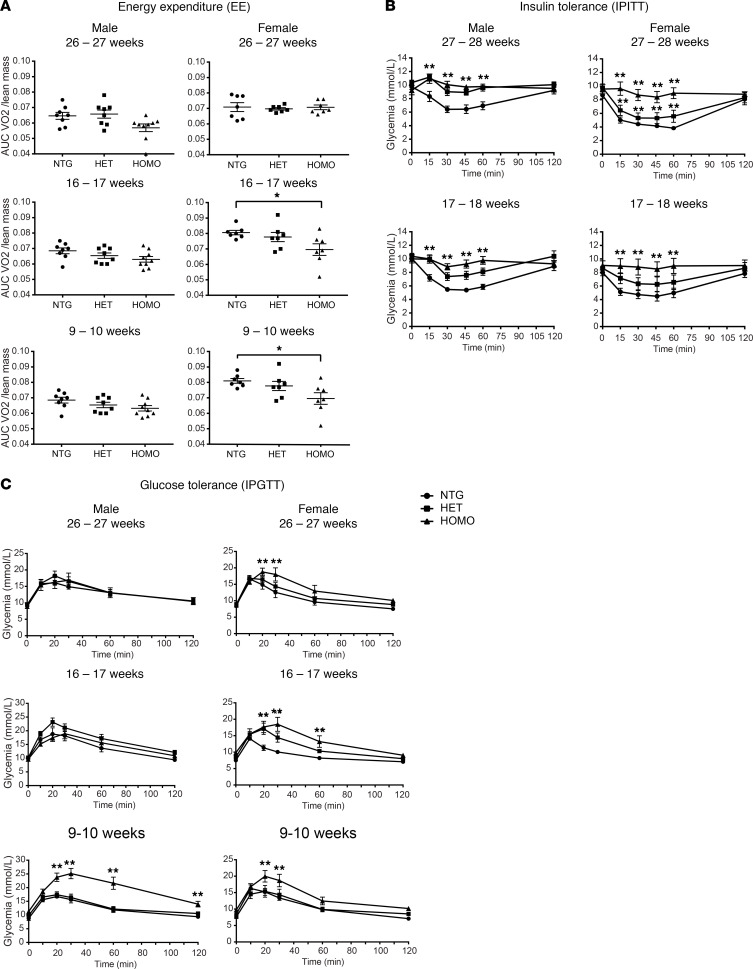
Energy expenditure, glucose tolerance, and insulin tolerance tests on R165W-hMC4R-KI mice and their control littermates. (**A**) Oxygen consumption (*V*O_2_) and carbon dioxide production (*V*CO_2_) were evaluated every 15 minutes over 24 hours on individualized R165W-hMC4R-KI male (left) or female (right) mice. Energy expenditure data are represented as AUC/lean mass (9- to 10-, 16- to 17-, and 26- to 27-week-old, males: NTG = 8, HET = 8, HOMO = 9; females: NTG = 7, HET = 7, HOMO = 7). (**B**) Insulin tolerance was measured on R165W-hMC4R-KI male (left) or female (right) mice injected i.p. with 0.75 U/kg of insulin after 6 hours’ fasting (17- to 18- and 27- to 28-week-old, males: NTG = 8, HET = 9, HOMO = 9; females: NTG = 7, HET = 8, HOMO = 7). (**C**) Glucose tolerance was measured on R165W-hMC4R-KI male (left) or female (right) mice injected i.p. with 1 g/kg of d-glucose after 12-hour fasting (males: 9- to 10-, 16- to 17-, and 26- to 27-week-old NTG = 8, HET = 9, HOMO = 9; females: 9- to 10-week-old NTG = 6, HET = 8, HOMO = 6, 16- to 17- and 26- to 27-week-old NTG = 7, HET = 8, HOMO = 7). Data are the mean ± SEM and were analyzed using 1-way Kruskal-Wallis test of variance followed by pairwise comparisons using post hoc Dunn’s multiple comparisons test (**A**) or 2-way ANOVA test of variance followed by pairwise comparisons using post hoc Bonferroni’s multiple comparisons test (**B** and **C**). The asterisks denote significant difference of either HOMO or HET hMC4R-KI mice compared with NTG littermates. *P < 0.05, **P < 0.001.

Given the role of MC4R in the adaptive metabolic response to excess caloric consumption, we monitored energy expenditure by measuring 24-hour O_2_ consumption (*V*O_2_) in male and female R165W-hMC4R mice at different ages (Figure 5A). The data were expressed as a function of the lean mass to consider the increased lean mass observed in the MC4R-deficient mice compared with their nontransgenic littermates (28). No significant change in *V*O_2_ consumption was observed in either R165W-hMC4R heterozygous or homozygous obese male mice when compared with their nontransgenic age-matched littermates. In females, no difference was observed for heterozygous R165W-hMC4R mice, but a reduced *V*O_2_ consumption was found for homozygous R165W-hMC4R females at 9 and 16 weeks of age. These data indicate that R165W-hMC4R mice are not able to adjust their energy expenditure to their increased food intake, most likely contributing to their obese phenotype, as previously observed for MC4R-KO mice (31).

The ability of the R165W-hMC4R mice to regulate their glycemia was also assessed using glucose and insulin tolerance tests. As shown in Figure 5B, an impaired response to insulin injection was observed in both male and female homozygous R165W-hMC4R mice measured at 17–18 and 27–28 weeks, indicative of insulin resistance. When considering the glucose tolerance test, whereas a sustained hyperglycemia was observed at all ages tested for the female homozygous R165W-hMC4R mice, such significant hyperglycemia was observed only at 9 weeks of age for the males, their response to glucose returning to normal at 16–17 and 26–27 weeks of age (Figure 5C). These data suggest that mice carrying the R165W allele are hyperglycemic most likely because of developing insulin resistance, which is consistent with the phenotype previously reported for MC4R-null mice (31).

### Characterization of the PC candidate UM0130866.

**Figure 6:**
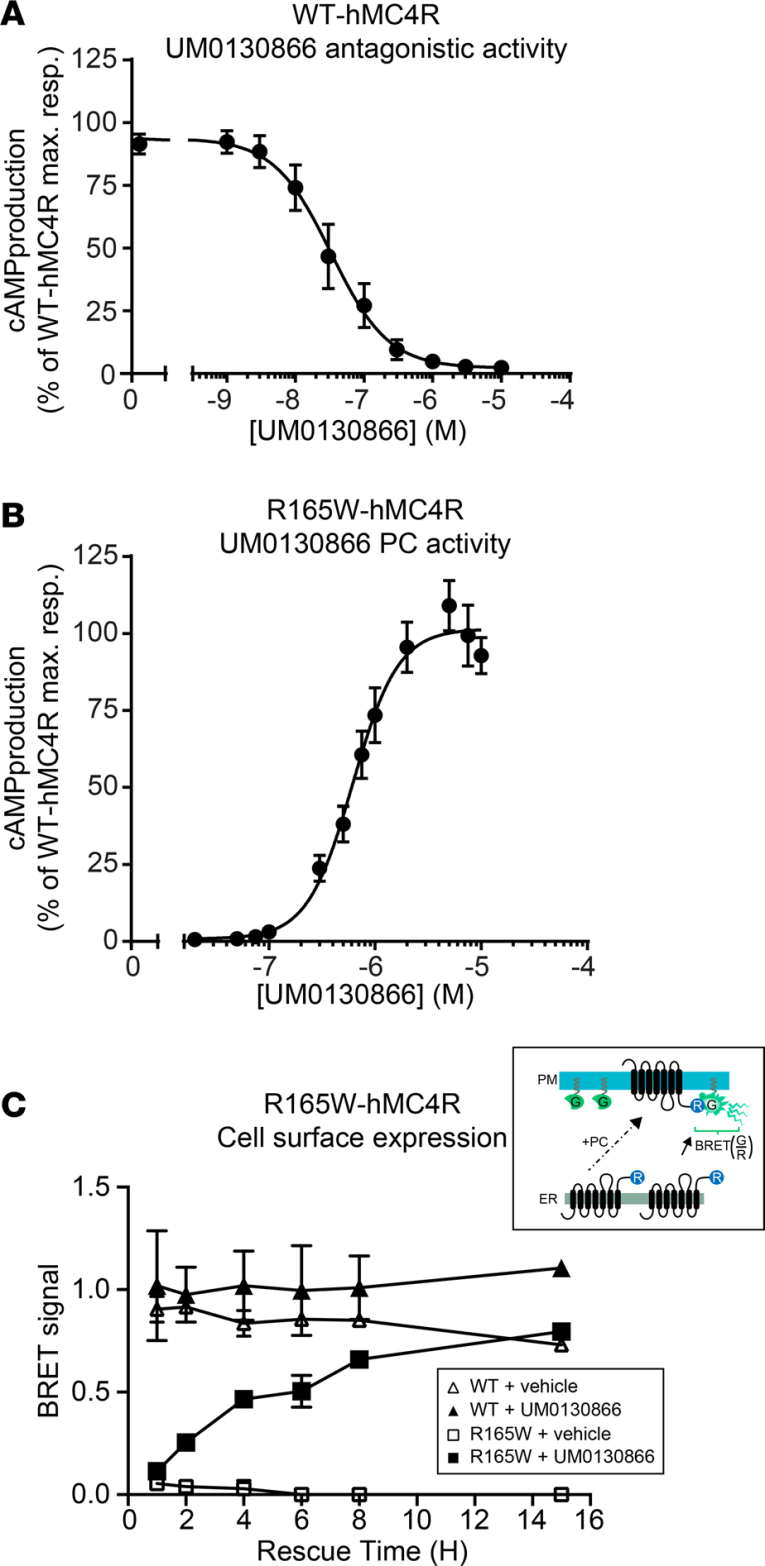
Characterization of PC UM0130866 compound. (**A**) Evaluation of antagonist potency of compound UM0130866 on WT-hMC4R. HEK293T cells stably expressing the WT-hMC4R were preincubated for 1 hour with increasing concentrations of compound UM0130866, followed by the addition of 25 nM NDP-α-MSH, concentration corresponding to EC_50_, for 30 minutes. cAMP accumulation was then measured using a competitive immunoassay based on homogeneous time-resolved fluorescence (HTRF) technology (cAMP dynamic-2 kit from cis-bio). Data are expressed as percentage of inhibition compared with maximal cAMP response of WT-hMC4R–expressing cells upon 25 nM NDP-α-MSH exposure. Data are the means ± SEM of 5 independent experiments. (**B**) Evaluation of PC potency on R165W-hMC4R. HEK293T cells stably expressing the R165W-hMC4R were treated with increasing concentrations of UM0130866 for 15 hours. Cells were then washed and incubated 1 hour at 37°C with 3 μM of NDP-α-MSH and cAMP accumulation was measured. Data are expressed as percentage of activation compared with maximal cAMP response of WT-hMC4R–expressing cells upon a saturated concentration for NDP-α-MSH (3 μM) in basal condition. Data are the means ± SEM (*n* = 7). (**C**) Effect on trafficking to the cell surface of WT- and R165W-hMC4R. HEK293T cells transiently expressing WT- (triangle) or mutant R165W- (square) hMC4R, tagged at their C-terminal with RlucII were incubated in the absence (open symbols) or presence (filled symbols) of 10 μM UM0130866 over time. Cell surface expression level was then measured by BRET between the receptor-RlucII (donor of energy) and rGFP-CAAX (acceptor of energy anchored at the cell surface). Inset: schematic representation of the cell surface expression BRET assay. Data are the mean ± SD of 2 independent experiments.

To test the therapeutic potential of the PC approach in the context of MC4R deficiency, we selected a PC candidate, UM0130866, identified by screening of a compound library that was optimized by medicinal chemistry in collaboration with Pfizer scientists and the medicinal chemistry platform of IRIC. As shown in Supplemental Table 2, UM0130866 binds to MC4R with high affinity (inhibitory constant, Ki: 73 nM) and a selectivity of 245-, 16-, and 8-fold over MC1R, MC3R, and MC5R, respectively. When tested on the ability of WT-hMC4R to promote cAMP production in response to [Nle4,D-Phe7]–alpha-MSH (NDP-α-MSH), UM0130866 was found to be a full antagonist with a half maximal inhibitory concentration of 33 nM (Figure 6A). The PC activity of this MC4R-selective antagonist in cell-based assays was illustrated by its ability to rescue cell surface expression of R165W-hMC4R (Figure 6C). This rescued cell surface expression of R165W-hMC4R was accompanied by a complete restoration of receptor-mediated cAMP production upon NDP-α-MSH stimulation (Figure 6B). The potency (EC_50_) of UM0130866 to act as a PC to rescue MC4R signaling was found to be 441 nM.

**Figure 7:**
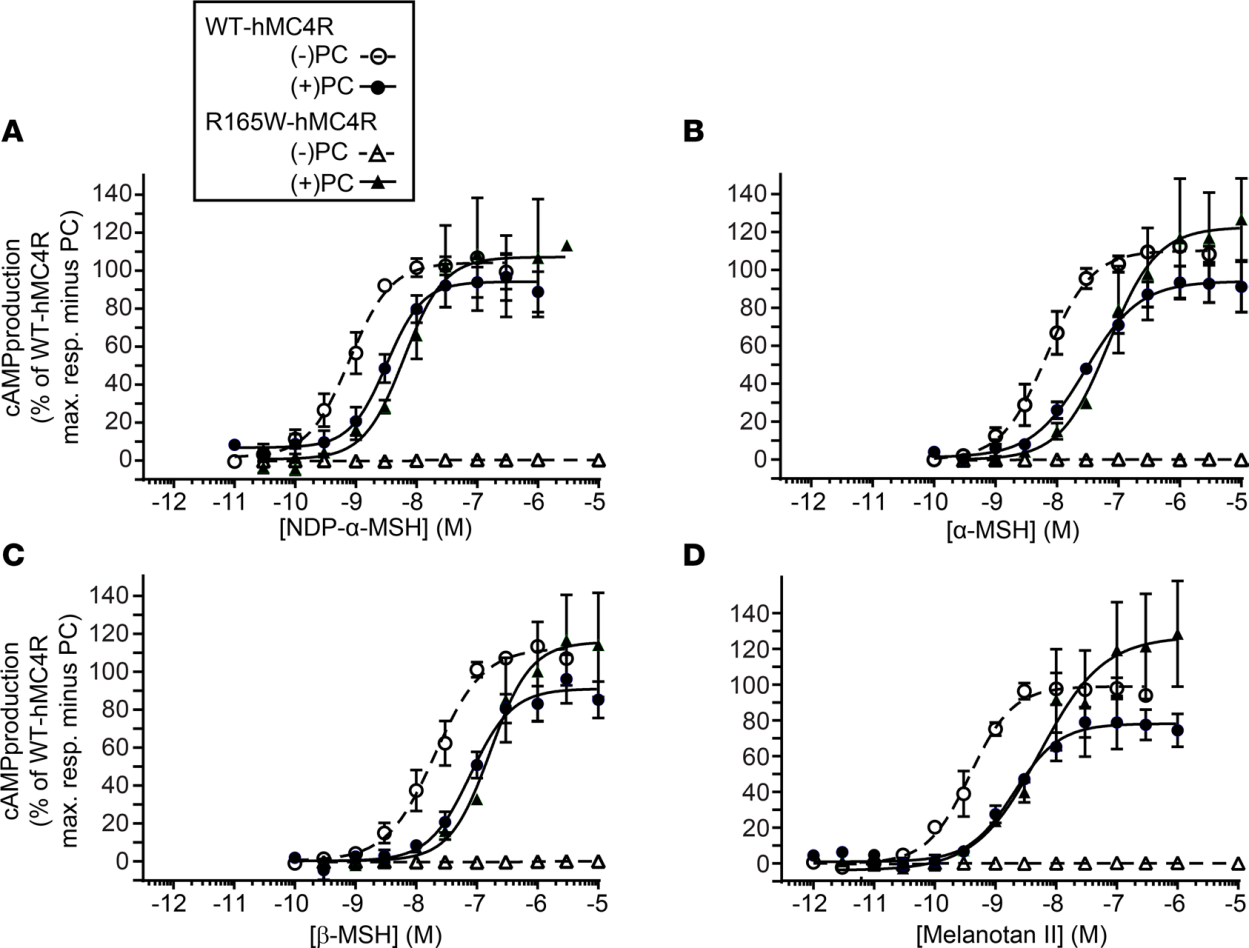
Evaluation of melanocortin agonist potencies on WT-hMC4R and R165W-hMC4R in the presence or absence of UM0130866 (PC). Cells expressing either the WT-hMC4R or R165W-hMC4R were treated (+) or not (-) with 10 μM UM0130866 for 15 hours. Cells were then washed in 1× Dulbecco’s PBS (D-PBS), pH 7.4, and incubated 1 hour at 37°C with increasing concentrations of melanocortin agonists NDP-α-MSH (**A**), α-MSH (**B**), β-MSH (**C**), or MTII (**D**). cAMP accumulation was then measured using a competitive immunoassay based on HTRF technology (cAMP dynamic-2 kit from cisbio). Data are expressed as percentage of cAMP response for WT-hMC4R upon stimulation with a saturating concentration of NDP-α-MSH (3 μM) under basal condition. Data are the means ± SEM of 3 independent experiments.

We also assessed the pharmacological properties of the R165W-hMC4R mutant receptor rescued by the PC. The cAMP responses (potency and efficacy) to natural and synthetic melanocortin agonists, NDP-α-MSH, α-MSH, β-MSH, and MTII, on the rescued R165W-hMC4R following treatment with UM0130866 were comparable to those measured for the WT-hMC4R after PC treatment. However, the dose-response curves were slightly right shifted (4- to 5-fold) compared with untreated WT-hMC4R (Table 1 and Figure 7), most likely reflecting residual UM0130866 antagonist activity.

To further dissect the dynamic process of the R165W-hMC4R rescue by UM0130866 and to determine the time of exposure needed to fully recover the maximum expression level of the mutant receptor at the cell surface, we performed a BRET-based assay monitoring receptor targeting to the plasma membrane (34). UM0130866 promoted a time-dependent rescue of the R165W-hMC4R cell surface targeting, reaching after 8-hour treatment 88% of the value observed for the untreated WT receptor (Figure 6C).

Taken together, these data indicate that the MC4R antagonist, UM0130866, acts as a very efficacious PC with reasonable potency and restores WT-like signaling properties to R165W-MC4R in cell-based assays, making it a candidate for in vivo testing.

### Treatment with PC UM0130866 rescues melanocortin sensitivity in R165W-hMC4R-KI mice.

To determine whether UM0130866 has pharmacokinetic properties compatible with its use in vivo, free concentration and the time of exposure of UM0130866 formulated as spray-dried dispersion (SDD; to enhance bioavailability) were determined in R165W-hMC4R homozygous mice (Supplemental Figure 3). For the 2 highest oral doses tested (60 mg per kg [mpk] and 100 mpk), the free maximum plasma concentration levels were respectively 2.2- and 4-fold over the unbound fraction cell-based PC potency (≈250 nM represented by the dashed line in Supplemental Figure 3). At the highest dose administered (100 mpk), the free fraction concentration of UM0130866 remained at 2-fold over the in vitro PC potency of unbound fraction for up to 6 hours, a kinetic pattern favorable for rescuing a significant amount of mutant receptor at the cell surface.

**Figure 8:**
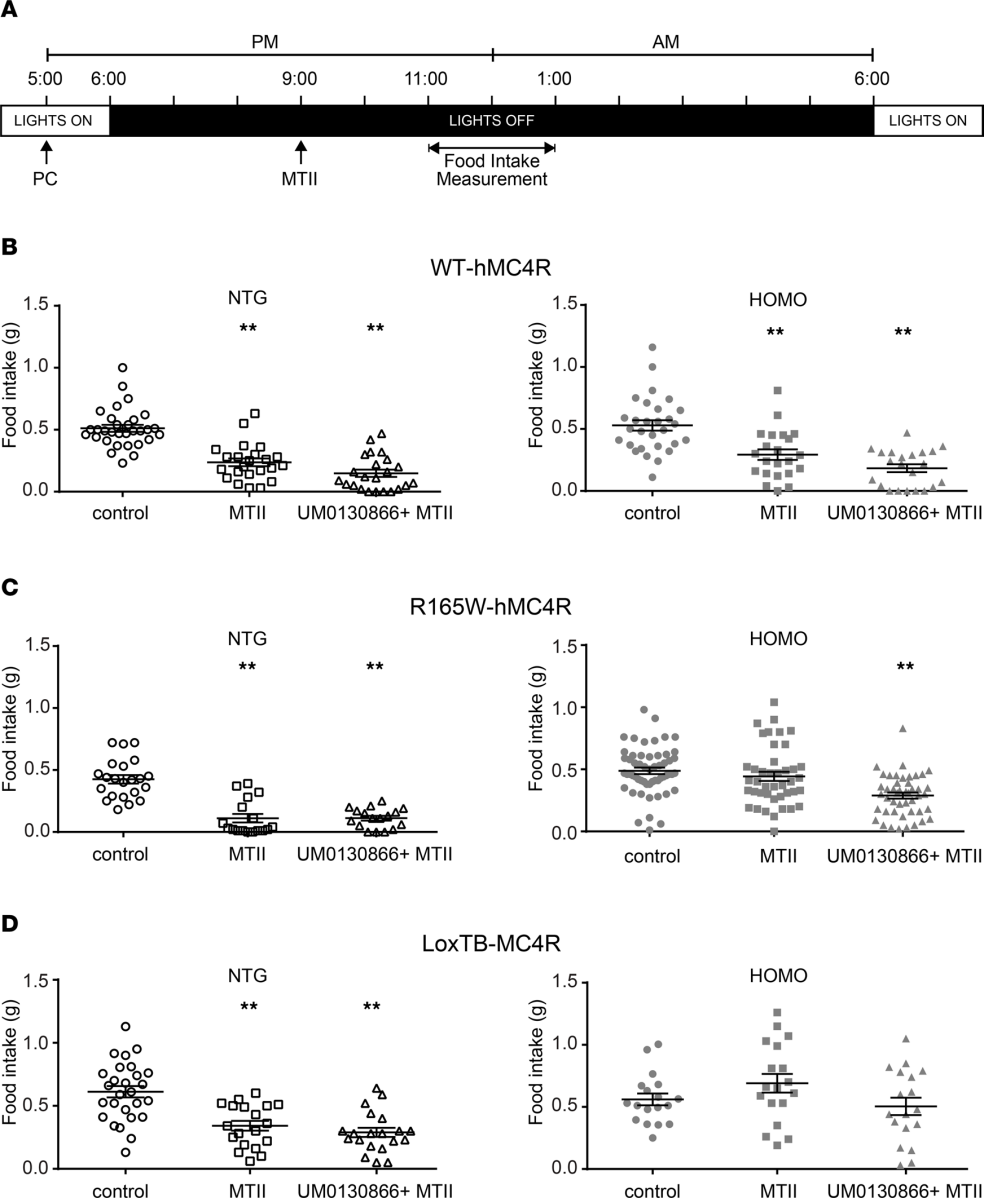
Effect of UM0130866 on MTII agonist response on food intake in hMC4R-KI mice and in loxTB MC4R-null mice. (**A**) Schematic representation of the paradigm. (**B**–**D**) HOMO male mice from each transgenic line at 13 to 18 weeks old and their NTG littermates, respectively, received per os (gavage) 100 mpk UM0130866: SDD formulated in 0.5% methyl cellulose (metcell) in water, at ZT11 (1 hour before lights off) followed by an i.p. injection of 10 mpk (NTG) or 20 mpk (Homo) of MTII at ZT15 (3 hours after lights off) using a randomized crossover experimental design. Food intake was measured for 2 hours between ZT17 (5 hours after lights off) and ZT19 (7 hours after lights off) in individualized mice. Data are the mean ± SEM of WT-hMC4R-KI mice (**B**) (NTG = 23 and HOMO = 22), R165W-hMC4R (**C**) (NTG = 17; HOMO = 45) and loxTB MC4R-null mice (**D**) (NTG = 19; HOMO = 18). The asterisks denote significant difference of treated group compared with control group using 1-way Kruskal-Wallis test of variance followed by pairwise comparisons using post hoc Dunn’s multiple comparisons test. **P < 0.001.

To test the in vivo PC efficacy of UM0130866, we assessed its ability to restore sensitivity to the melanocortin agonist MTII stimulation in homozygous R165W-hMC4R-KI mice using food intake as readout (Figure 8A). As previously observed in Figure 1C, MTII promoted a significant decrease in food intake in nontransgenic but not in R165W-hMC4R animals (Figure 8C). Treatment with UM0130866 before MTII administration led to a decrease in food consumption of 40% compared with their basal food intake in control conditions, showing that prior exposure to UM0130866 restores the anorexic effect of MTII in homozygous R165W-hMC4R mice (Figure 8C). In contrast, no significant effect of the PC treatment was observed on the anorexic response of MTII in the WT-hMC4R homozygous mice (Figure 8B). No interference on MTII response, such as negative competition, was seen in mice with a functional receptor (i.e., in nontransgenic littermates or in homozygous WT-hMC4R mice), showing that MTII was able to displace the PC antagonist. Furthermore, no change in food intake was observed in homozygous loxTB MC4R null mice, either upon MTII only or following the cotreatment (UM0130866 + MTII) (Figure 8D), demonstrating that the anorexigenic effect observed in homozygous R165W-hMC4R mice was dependent on functional rescue of hMC4R, consistent with the PC action of the compound. Altogether, those data provide the first in vivo proof of principle to our knowledge that the PC approach is efficient in partially reversing the MC4R deficiency–linked food intake effects.

## Discussion

This study describes 2 potentially unique humanized MC4R-transgenic mouse models harboring either a WT or a mutant human allele in place of the mouse receptor allele. Both models are MC4R dependent and recapitulate many of the characteristics of human obesity resulting from deficiencies in the melanocortin system, such as increased food intake, weight gain, hyperglycemia, and increased linear growth. However, the mechanisms leading to the obesity phenotypes are clearly different for the 2 models. As expected for a loss-of-function mutation resulting from receptor misfolding, mice harboring 2 copies of the R165W-hMC4R allele did not respond any more to central or peripheral administration of MTII melanocortin agonist, consistent with the notion that the lack of receptor expression at the cell surface is the primary cause leading to obesity. In contrast, obese mice harboring the WT-hMC4R alleles remained fully responsive to the synthetic agonist MTII and the agonist β-MSH and showed decreased sensitivity to α-MSH, resulting in a hypomorphic phenotype. The study is also the first in vivo proof of concept to our knowledge that a PC can rescue the function of a misfolded obesity-causing hMC4R-mutant allele. In addition to opening new therapeutic possibilities for this disease, the data show that the R165W-hMC4R mice represent a valid model to test the in vivo therapeutic potential of PCs.

When considering the WT-hMC4R-KI obesity model, the difference in sensitivity to α-MSH is dependent on the species of the receptor and not on a difference in the primary sequence of α-MSH, since mouse and human sequences are perfectly conserved. In contrast, we did not observe such difference in sensitivity to β-MSH, which is produced in humans but not in mice (32, 33), suggesting that the difference in α-MSH sensitivity most likely does not reflect a difference in the expression level of the transgenic receptor compared with the mouse MC4R and reinforcing the idea that β-MSH plays a significant role in energy homeostasis in humans. The potential involvement of β-MSH in human body weight regulation has previously been revealed through the reports of heterozygous loss-of–function mutations occurring in the *POMC* gene identified in obese people (35–37). More recently, another example of the β-MSH implication in the maintenance of energy homeostasis in mammals was reported, describing a frameshift mutation in the coding sequence of the canine POMC gene leading to obesity (38). Taken together, these data underline the significance of β-MSH secretion in maintaining energy homeostasis in mammals that produce it and that the hypomorphism observed in mice carrying the WT-hMC4R allele might result from a decrease in melanocortin tone due to the absence of β-MSH in mice, leading to the development of an obese phenotype. Moreover, from an evolutionary point of view, the reduced sensitivity of the hMC4R to α-MSH compared with mouse receptor is consistent with the apparent predominance of the β-MSH system in humans that is also illustrated by the detection of a higher number of β-MSH–positive neurons compared with α-MSH neurons in human arcuate nucleus (35).

Consistent with the hypothesis that a decrease in melanocortin tone is the main cause of the WT-hMC4R-KI mice’s hypomorphic phenotype, we observed that chronic central infusion of the superagonist MTII (39) restored a near normal cumulative food intake in homozygous WT-hMC4R mice (Figure 4A) and induced a significant weight loss (Figure 4C). The fact that homozygous WT-hMC4R mice are sensitive to MTII chronic exposure indicates that the hypomorphic phenotype results from a decrease in MC4R agonist tone rather than a fault in the transgenic human WT receptor sensitivity.

It follows that we have generated 2 types of obese mouse models, one carrying a hypomorphic WT-hMC4R allele in mouse background, which might be useful in the context of studying melanocortin deficiency and a second one carrying an amorphic mutant allele corresponding to a model mimicking a conformational disease. The mouse harboring the amorphic R165W mutant allele represents a good model for the MC4R-linked familial early-onset obesity as it mimics many of the characteristics observed in humans with MC4R loss-of-function mutations, such as hyperphagia, increase in linear growth, early-onset obesity, as well as symptoms of type 2 diabetes such as elevated glycemia (3). R165W is a mutation reported in several obese patient cohorts (19–23) and is representative of a large number of obesity-causing MC4R mutations resulting from misfolding and intracellular retention of the receptor.

The observation that UM0130866 acute treatment restored the anorexigenic response of R165W-hMC4R-KI mice to MTII provides a proof of principle that the PC approach represents a promising therapeutic avenue for the treatment of obesity resulting from misfolded MC4R and demonstrates that this potentially new MC4R mouse model is a valid in vivo tool to test PC activity.

Although this is likely the first in vivo proof of principle for the action of an MC4R PC, a previous study has demonstrated that PCs targeting the GnRHR can restore the activity of a mouse model in which a mutation responsible for hypogonadotropic hypogonadism was introduced in the mouse GnRHR gene (11), illustrating the generalizability of the concept.

One particularity of the model presented here is the introduction, in the mouse, of the human gene harboring a disease-causing mutation, increasing the model’s drug discovery translational value and possibly unmasking species differences. This latter aspect is clearly illustrated by the unexpected hypomorphic phenotype observed for the WT-hMC4R-KI mice that revealed the lower sensitivity of the human MC4R to α-MSH, which, combined with the lack of β-MSH in mouse, resulted in obesity. Such lower responsiveness of the human versus mouse MC4R to α-MSH is most likely counterbalanced in humans by the presence of both α-MSH and β-MSH.

One of the implications of the R165W-hMC4R functional rescue by UM0130866 is that PCs with antagonistic properties could be therapeutically relevant if the pharmacokinetics (PK) of the compound allow its dissociation from the receptor to enable the agonist action once the receptor has been chaperoned to the cell surface. Such favorable PK have also been seen for the GnRHR-targeting PC discussed above (11) as well as in the case of PCs targeting the vasopressin V2 receptor for the treatment of nephrogenic diabetes insipidus in humans (12).

Yet, the design of long-term exposure dosing needed for therapeutic purposes might be challenging considering the pulsatile pattern required in this strategy and the circadian release profile of melanocortins. New compounds with the required PC potency and PK profiles will therefore be needed to address the efficiency of such a therapeutic approach on reversing the obesity phenotype.

In recent studies, a superagonist, setmelanotide/RM-493, was successfully tested in obese patients with MSH deficiency as hormone replacement therapy (40). In a separate study, the therapeutic potential of this compound was examined in a cohort of heterozygous MC4R-deficient patients (41). Although no significant weight loss was observed compared with placebo treatment, a modest weight loss was seen in some patients. The authors suggested the intriguing possibility that setmelanotide/RM-493 could act as a weak PC. However, given the peptidic nature of the compound, its action as a PC, requiring intracellular penetration, is unlikely. Alternatively, the presence of only 1 functional WT allele may be sufficient to provide a response to a superagonist such as setmelanotide/RM-493. Further studies are needed to establish the mechanism by which this compound could have an effect on some heterozygous MC4R-deficient patients.

A PC-based approach such as the one described in this study could theoretically be efficient for both homozygous and heterozygous patients as it rescues the mutated allele and does not rely on the presence of a functional allele. However, it would be predicted that PCs would be more efficacious in heterozygous patients since the amount of rescued receptors needed to attain the anorexic response threshold would be more easily reached given the residual activity provided by the WT allele. It could also be an effective approach even if some intracellularly retained mutants had dominant negative effects by interfering with the cell surface trafficking of the WT allele, as has been shown for MC4R and other MC receptors (42, 43).

This is an important point since most patients have heterozygous deficiencies. It was previously found that the administration of MC4R agonists could have adverse cardiovascular effects resulting from the sustained activation of the Gq pathway (44). UM0130866 being an antagonist, it would presumably not activate this pathway. Moreover, the PC strategy relying on the activation of the rescued receptor by its endogenous agonists, which would be secreted under normal spatiotemporal conditions, would reduce the possible on-target adverse cardiovascular impact that an exogenous agonist could have. It should also be reemphasized that effectiveness of the treatment would be dependent on the PK of the compound and the dosage regimen used to allow binding of endogenous agonists. Intermittent (pulsatile) PC administration would provide PC-free periods during which PC dissociation occurs, and a rescued receptor can be active as has been shown for rescuing a mutant form of GnRHR in an in vivo mouse model (11).

The present study clearly demonstrated that PC treatment could restore the anorexic response of homozygous R165W-hMC4R mice to acute treatment with melanocortin receptor agonist. However, the impact of chronic PC treatment on weight loss remains to be investigated. The lower responsiveness of the human receptor to α-MSH may require that preclinical studies carried out in mice include cotreatment with exogenous ligands that display high affinity for hMC4R, such as β-MSH or MTII. This mutation will not affect clinical studies since β-MSH is endogenously produced in humans. In addition to food intake and weight gain, biomarkers of PC actions could include the development of positron emission tomography probes to detect the rescue of receptor binding site in the hypothalamus. Additionally, circulating levels of peptide YY could be used as a biomarker for MC4R activation, as its release has been proposed under the control of MC4R (45).

Given the limited therapeutic options for severely obese patients harboring MC4R mutations, alternative strategies targeting the molecular defect responsible for the disease may address an unmet medical need. The proof of principle presented in the present study is an important step toward testing PC candidates that may represent such an alternative strategy for the treatment of MC4R-linked familial early-onset obesity.

## Methods

### Production of knockin mice and animal care

Using standard ES cell procedures (see Supplemental Methods for details), chimeric animals were obtained and mated with C57BL/6 mice to generate mice heterozygous for either the 3HA-hMC4R(R165W)-Venus or myc-hMC4R(WT)-Venus allele on a mixed C57BL6/J (The Jackson Laboratory) and 129S6B6F1 (ES cells) background. Animals were housed under specific pathogen–free conditions in ventilated cages and were handled in accordance with procedures and protocols approved by Université de Montréal and Université Laval institutional animal care committees. Prior to experiments, mice were housed in groups of 2 to 5 mice at 22°C–24°C using a 12-hour light/12-hour dark cycle (6 am to 6 pm) and fed ad libitum with regular chow (Teklad global 18% protein diet 2028, 3.1 kcal/g metabolizable energy, 18% kcal from fat, Harlan Teklad) with water provided ad libitum. Heterozygous mice were backcrossed 3 times on C57BL6/J genetic background and were then maintained by breeding heterozygotes inter se.

LoxTB MC4R heterozygous mice, carrying a loxTB sequence that prevents normal endogenous MC4R transcription and translation from the endogenous locus, were purchased from The Jackson Laboratory (catalog 006414) (24, 39), to be used as negative controls, and were bred in the same conditions and facilities as described for the humanized MC4R mouse lines.

### Immunohistochemistry

#### Anti-GFP immunolabeling.

Brains from transgenic animals were collected, snap-frozen in isopentane, and stored at –80°C until further processing. Sections were cut at 10 μm using a cryostat. Serial 10 μm thick frozen brain sections were then processed in the automatic immunostainer Discovery XT system (Ventana Medical Systems, Roche) for indirect peroxidase labeling using anti-GFP (ab290 from Abcam at 1:1000) as primary antibody against the C-terminal yellow fluorescent protein fused to the transgene receptor. Streptavidin horseradish peroxidase and 3,3-diaminobenzidine were then used according to the manufacturer’s instructions (DABmap detection kit, Ventana Medical Systems, Roche). Tissue sections were then stained using a conventional hematoxylin and eosin protocol. Finally, each slide was coverslipped and scanned at high resolution (original magnification, 40×) using the Nanozoomer Digital Pathology equipment (Hamamatsu).

#### Anti-MC4R immunolabeling.

Brains from transgenic animals were harvested and maintained in paraformaldehyde (4%) for 7 days and transferred into a solution containing paraformaldehyde (4%) and sucrose (10%) until further processing. Brain sections (25 μm) were cut from the olfactory bulb to the brainstem using a sliding microtome (MICROM International GmbH, model HM 440E). Brain slices were stored at –30°C in a cryoprotective solution containing sodium phosphate buffer (50 mM), ethylene glycol (30%), and glycerol (20%) and mounted on poly-l-lysine–coated slides. Mouse brain coronal sections (7 μm) were sequentially incubated at room temperature in methanol containing 3% of H_2_O_2_ (30 minutes), PBS containing 0.1% of Triton X-100 (10 minutes), 0.25% trypsin (2 minutes, 37°C), and PBS containing 1% of normal goat serum for 1 hour. They were then incubated at 4°C for 18 hours with rabbit affinity-purified antiserum directed against MC4R (1:400; Alamone). Sections were rinsed with PBS, incubated with SignalStain Boost IHC detection reagent (Cell Signaling Technology, 30 minutes, room temperature, RT) and labeled using SignalStain DAB substrate kit (Cell Signaling Technology). Coronal sections were rinsed with PBS and incubated with DAB/nickel peroxidase substrate (Vector Laboratories). After washes in PBS, slices were mounted in Distyrene Plasticizer Xylene. Images were captured on Olympus BX51 microscope using QImaging camera (model Retiga 2000R) and Image-Pro Plus 7.0 software (Media Cybernetics).

### Phenotyping

At 3 weeks of age, mice were weaned and group-housed with littermates of the same sex. At 4 weeks of age, weight gain was measured regularly on a weekly basis until 15 weeks of age using a Sartorius ELT402 balance. Length was measured on anesthetized mice (short induction with isoflurane gas) at 16–17 weeks old by manual immobilization and extension of the mice to their full length and measurement of the nose-to-anus distance in centimeters. Whole blood was collected by tail vein bleeding in fasted mice (5 hours fasting prior to bleeding) at 16–17 weeks of age, and blood glucose was assessed using Accu-check Aviva meter (Roche). Basal food intake on regular chow diet (Teklad Global 18% Protein Rodent Diet from Harlan Laboratories) was measured in individualized mice of 19–20 weeks of age. Mice were individually housed at least 4 days before any measurements were taken. A sufficient amount of food for the week was then weighed and provided to the mice ad libitum. Each day, morning (8 am) and afternoon (5 pm) at the same time, the remaining food was measured for 5 consecutive days. The daily average of food intake during dark cycle and light cycle was calculated for each genotype. The caloric intake was estimated using 3.1 kcal/g as metabolizable energy.

### Icv injections

#### Stereotaxis.

Adult male mice from each transgenic line (loxTB MC4R-null mice, hMC4R-KI [WT or R165W] mice) were stereotaxically implanted with a permanent 22-gauge single-guide cannula (PlasticsOne) aimed at the lateral ventricle using the following stereotaxic coordinates: 0.4 mm posterior to the bregma, 1 mm lateral to the bregma, and 2 mm ventral to the skull surface. The guide cannulas were secured with screws and cranioplastic cement (Dentsply Canada). To prevent clogging and to reduce the potential for brain infection, sterile obturators (PlasticsOne) were inserted into the guide cannulas. Appropriate cannula placement was functionally verified before experiments using the dipsogenic effects of angiotensin 2 injections (5 ng) as positive control, 5 days after surgery.

#### Melanocortin agonist sensitivity measurement.

The day prior to agonist sensitivity assay, basal food consumption was measured individually between Zeitgeber time 10 (ZT10) (10 hours after lights on) and ZT15 (3 hours after lights off). The days of treatment, mice were icv injected with MTII (1 nM), β-MSH (20 pM; 100 pM), or α-MSH (20 pM; 100 pM) in a final volume of 2 μL of aCSF (Harvard Apparatus), at ZT10. Food consumption was then assessed between ZT10 and ZT15. In order to evaluate the impact of each treatment, food consumption was then normalized relative to the basal food consumption for each mouse. The sequence of the α-MSH peptide used is shared between mouse and human whereas the human sequence was used for β-MSH since it is not produced in the mouse.

### Subchronic infusions with osmotic minipump implantation

MTII agonist or aCSF (vehicle) was infused icv at a dose of 1 nmol/d in the lateral ventricle using 22-gauge single-guide cannula (PlasticsOne) aimed at the lateral ventricle (as previously described in *Stereotaxis*) and connected by a catheter to an Alzet osmotic minipump (model 1002). The pumps, which deliver their content at a flow rate of 0.25 μL/h for 14 days, were subcutaneously implanted in the interscapular region. Body weight and food consumption were evaluated each day at ZT6 (6 hours after lights on) on individualized mice during 1 week.

### Indirect calorimetry experiments

Mice were placed in metabolic chambers (AccuScan Instruments, Inc.) and acclimated for 72 hours with free access to food and water. Then, *V*O_2_ and *V*CO_2_ were evaluated every 15 minutes over 24 hours in an open circuit system with an oxygen analyzer (Applied Electrochemistry, S-3A1) and carbon dioxide analyzer (Applied Electrochemistry, CD-3A) as previously described (46). Energy expenditure data are represented by AUC/lean mass as differences have been observed in lean mass between groups.

### Glucose tolerance test and insulin tolerance test

For the i.p. glucose tolerance test, mice were fasted for 12 hours and were injected i.p. with 1 g/kg of d-glucose (MilliporeSigma). For the i.p. insulin tolerance test, animals were fasted for 6 hours and were injected i.p. with 0.75 U/kg of insulin (Humulin, Lilly). Blood samples were collected from the tail vein, and glucose was measured using a glucometer (OneTouch).

### Cells for in vitro assays (cAMP and BRET)

HEK293T cells (ATCC) stably (for cAMP accumulation) or transiently (BRET) expressing hMC4R were used for cell-based assays. Cells were regularly tested for mycoplasma contamination (PCR Mycoplasma Detection kit, abm), and only mycoplasma-negative cells were used for the assays.

### cAMP assay

Intracellular cAMP accumulation was measured using a competitive immunoassay based on homogeneous time-resolved fluorescence technology (cAMP dynamic-2, cisbio). hMC4R HEK293T stable cell lines expressing either the WT or mutant form R165W 3HA-hMC4R-Venus constructs were dispensed in 96-well plates (15 × 10^3^ cells/well) and processed as described below.

For antagonist potency assessment, cells were preincubated with increasing concentrations of UM0130866 for 1 hour at 37°C on a Heidolph Titramax 100 shaker at speed 900 rpm in complete buffer: 140 mM NaCl, 2.7 mM KCl, 1 mM CaCl_2_, 12 mM NaHCO_3_, 5.6 mM d-glucose, 0.49 mM MgCl_2_, 0.37 mM NaHPO_4_, 25 mM HEPES at pH 7.4, 0.75 mM 3-isobutyl-1-methylxanthine, 0.01% BSA (*w/v*). Then, 25 nM of NDP-α-MSH (corresponding to EC_50_) in complete buffer was added for 30 minutes at 37°C as above. For maximal response (100%), cells were only incubated with 25 nM NDP-α-MSH as described above. Cells were kept on ice for 5 minutes before proceeding to cAMP measurement. For PC potency assessment of UM0130866, cells were treated the day before the assay with increasing concentrations of UM0130866 for 15 hours. After wash in 1× D-PBS (pH 7.4), cells were incubated 1 hour at 37°C on Heidolph Titramax 100 shaker at speed 900 rpm in complete buffer containing 1 μM NDP-α-MSH. For agonist potency measurement after PC treatment, cells were treated in the presence or absence of 10 μM UM0130866 for 15 hours before the cAMP assay, washed in 1× D-PBS at pH 7.4, and incubated 1 hour at 37°C on Heidolph Titramax 100 shaker at speed 900 rpm in complete buffer containing increasing concentrations of melanocortin agonists. For all assays, plates were then put at –80°C at least 90 minutes and thawed at RT on Heidolph Titramax 100 shaker at speed 1050 rpm. Cell lysates were then transferred to 384-well plates, lysed, and incubated with cAMP labeled with the dye d2 and anti-cAMP M antibody labeled with Cryptate (cAMP dynamic-2 kit, cisbio) according to the manufacturer’s protocol. Reading of the homogeneous time-resolved fluorescence signal was performed on an Artemis time-resolved fluorescence resonance energy transfer plate reader (Cosmo Bio USA).

### Cell surface expression BRET-based assay

Rescue of mutant MC4R cell surface expression was measured using a BRET-based biosensor assay monitoring receptor targeting to the plasma membrane, as previously described (27). Briefly, HEK293 cells were transiently transfected with WT or mutant 3HA-MC4R-RlucII (BRET donor) along with rGFP-CAAX (BRET acceptor). Cells were then exposed to 10 μM of UM0130866 for the indicated time, and BRET was measured on a Mithras LB940 Multimode Microplate Reader (Berthold), 5 minutes after the addition of the Rluc substrate, coelenterazine 400a at 2.5 μM final concentration, using filters set at 410 ± 35 nm (RlucII) and 515 ± 10 nm (rGFP). BRET ratio was determined by calculating the ratio of the light emitted by rGFP over the light emitted by RlucII and normalized to untreated WT-MC4R (expression set at 1).

### Acute in vivo administration of UM0130866

In order to measure food intake for 7 hours during the nocturnal cycle, we proceeded gradually to a shift of the standard light/dark cycle (6 am to 6 pm) to another 12-hour light/12-hour dark cycle but with 7 hours’ time difference (11 pm to 11 am) from the original cycle.

Male mice at around 6 weeks old underwent during 1 month a gradual adaptation of time difference at a frequency of 1-hour shift every 2 days until reaching 7 hours’ time difference from the original dark/light cycle. Mice were maintained on regular chow and water provided ad libitum. Mice were then individualized 2 weeks before starting the assay. To acclimatize the animals prior to the experiment, food intake was measured by weighing the food every 2 hours from ZT11 (11 hours after lights on) to ZT19 (7 hours after lights off). These measures were used to validate the effectiveness of the mice’s circadian rhythm synchronization to the new schedule. Homozygous male mice from each transgenic line at 13 to 18 weeks old and their nontransgenic littermates, respectively, received per os (gavage) 100 mpk UM0130866: SDD formulated in 0.5% methylcellulose in water, at ZT11 (2 hours before lights off), followed by an i.p. injection of 10 or 20 mpk of MTII at ZT15 (3 hours after lights off). The anorexic effect of MTII on food intake was measured for 2 hours between ZT17 (5 hours after lights off) and ZT19 (7 hours after lights off). A randomized crossover experimental design was chosen. Each mouse received all treatments with a washout period of 2 days between sets of experiments. Food intake measurements were assessed as described above during that period.

### Statistics

Cell-based assays were run in triplicates with a number of experiments per assay corresponding to *n* ≥ 2–3. For the in vivo experiment, mice were age-matched, weight-matched, and randomly assigned to the treatment groups using nontransgenic littermates as controls. We chose a randomized crossover experimental design with a 2-day washout period in order to reduce number of animals used in each experiment, with *n* > 4 mice per group to achieve statistical significance. Exclusion criteria were 20% weight loss (considering normal weight, i.e., 25–30 g), no recovery of daily food intake 24 hours posttreatment (considering again normal range, i.e., 3.5–4 g of food consumption).

Data are presented as means ± SEM. All statistical tests or curve fitting were performed using GraphPad Prism version 6.0h for MacOS X (GraphPad Software).

For experiments with 2 experimental groups (evaluating treatment effect on weight loss in chronic MTII exposure experiments), comparisons were made using the nonparametric Mann-Whitney *U* test. For experiments with more than 2 experimental groups, quantitative data were subjected to 2-way Kruskal-Wallis test of variance. Pairwise comparisons were then made using post hoc Dunn’s multiple comparisons test. For experiments with repeated measures along time, quantitative data were subjected to 2-way ANOVA test of variance with pairwise comparisons using post hoc Bonferroni’s multiple comparisons test. For experiments with paired matched values, such as the test of melanocortin sensitivity, quantitative data were analyzed with the Wilcoxon matched pairs test. Differences with *P* < 0.05 were considered statistically significant. All curve fitting was conducted by nonlinear regression analyses.

### Study approval

Animals were handled in accordance with procedures and protocols approved by Université de Montréal and Université Laval institutional animal care committees. Experimental procedures were revised and approved by the University of Montréal and Université Laval animal ethic committees.

## Author contributions

PR, DL, DR, and MB contributed to the conception of the project, the study design, and the interpretation of the results. PR and DL performed experiments and supervised as well as analyzed the experimental data. PR is listed first because she initiated and largely led the project. PR and MB wrote the manuscript. DL and DR assisted in the writing and reviewing of the manuscript. MB and DR were responsible for obtaining funding for the project.

## Supplementary Material

Supplemental data

## Figures and Tables

**Table 1:**
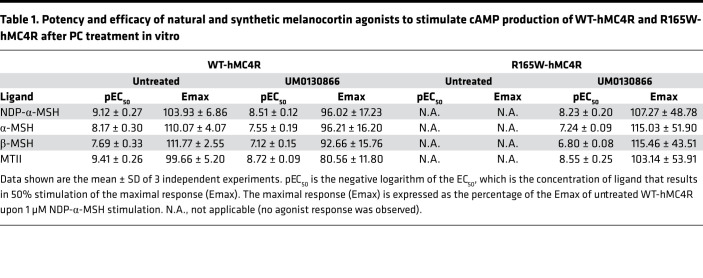
Potency and efficacy of natural and synthetic melanocortin agonists to stimulate cAMP production of WT-hMC4R and R165W-hMC4R after PC treatment in vitro

## Version 1 01/12/2021

In-Press Preview

## Version 2 02/22/2021

Electronic publication
