# Supplementary material for: Pharmacological chaperone action in humanized mouse models of MC4R-linked obesity
Source: JCI Insight. 2021 Feb 22;6(4):e132778. doi: 10.1172/jci.insight.132778 (PMC7934941; doi:10.1172/jci.insight.132778)
Supplement: Supplemental data [file jciinsight-6-132778-s061.pdf]

Supplemental for:

Pharmacological chaperone action in humanized mouse models of MC4R-linked obesity

Patricia René[1], Damien Lanfray[2], Denis Richard[2] and Michel Bouvier[1]

[1]*Départment de Biochimie et de Médecine Moléculaire, Institute for Research in Immunology and Cancer, Université de Montréal, 2950 chemin de Polytechnique, Montreal, Quebec, H3T 1J4, Canada.*

[2]*CRIUCPQ, Université Laval, 2725, Chemin Sainte-Foy, Québec, G1V 4G5 Canada.*

PR and DL have contributed equally to this work

D.R. and M.B. are co-corresponding authors

Address correspondence to: Michel Bouvier, Institut de Recherche en Immunologie et Cancérologie, Université de Montréal, 2950 chemin de Polytechnique, Montreal, Quebec, H3T 1J4, Canada. E-mail: michel.bouvier@umontreal.ca (M.B.), phone number: 514-343-6319 and Denis Richard, CRIUCPQ, Université Laval, 2725 Chemin Sainte-Foy, Québec, G1V 4G5, Canada. Email: denis.richard@criucpq.ulaval.ca (D.R.), phone number: 418-656-8711.

Supplemental Methods:

Mutagenesis and tag replacement

The mutant form of hMC4R (R165W) N-terminally tagged with 3xHA and containing an ApaI site in the coding sequence was generated by site-directed mutagenesis using overlap extension (1). This procedure involved two steps: 1) introduction of the desired base substitution into the hMC4R (WT) receptor cDNA using specifically designed complementary and overlapping primers, followed by 2) amplification of the mutated cDNA using the polymerase chain reaction (PCR). Each point mutation was inserted by PCR performed with Phusion taq polymerase (Finnzymes, NEB, Ontario, Canada) using specific primers containing the mutation complementary to opposite strands of the hMC4R (WT) and either a T7-Forward primer (5'-ATTAATACGACTCACTATAGGG-3') or a pcDNA3.1-Reverse primer (5'-AGAACGTGGACTCCAACGTCAAAG-3'). R165W Forward primer was 5'-G ACA GTT AAG **T**GG GTT GGG ATC ATC-3'. The first fragment was generated using the T7-Forward primer and the reverse/antisense primer complementary to forward sequence above. The second fragment was generated using the pcDNA3.1-Reverse primer and the forward/sense primer (sequence above). The WT form of hMC4R N-terminally tagged with myc and containing an ApaI site in the coding sequence was generated by PCR using the myc-BamHI-Bgl II Forward primer and the pcDNA3.1-Reverse primer. The myc-BamHI-Bgl II Forward primer has the following sequence: 5'-TCG GAT CCC CGA GAT CTC ACC ATG GCA TCA **ATG CAG AAG CTG ATC TCA GAG GAG GAC CTG** AAT TCG GT GAA CTC CAC CCA CCG T-3'. The 3xHA-hMC4R (WT) cDNA (Missouri S&T cDNA Resource center, USA) served as template in the PCR reaction to generate both the mutant form for 3HA-hMC4R(R165W) and the myc tagged WT form for myc-hMC4R(WT). Reaction conditions were 30 cycles of 94 °C (30 s), 55 °C (1 min), and 72 °C

(1 min). The fragments were then purified using the QIAGEN PCR purification kit (QIAGEN Mississauga, ON, Canada) and combined in the overlap extension reaction using T7-Forward and pcDNA3.1-Reverse primers described. Full length mutant PCR products were purified with QIAGEN gel extraction kit (QIAGEN Mississauga, ON, Canada) and inserted after restriction digest in KpnI/XhoI pcDNA3.1(+) vector. All PCR products were sequenced to confirm the presence of the desired mutation and absence of unwanted mutations.

Construction of 3HA-hMC4R(R165W) and myc-hMC4R(WT) modified to be compatible with the targeting vector

Plasmids described above were used as template to generate a 3HA-hMC4R(R165W) or a myc-hMC4R(WT) coding sequence containing a mutated Apa I site integrated in the targeting vector. BamHI and SacII restriction sites were inserted to be compatible with the targeting backbone vector. The following primers were used: 3HA-BamHI Forward: 5'-TAA GCT TGG ATC CAT GTA CCC ATA CGA TGT TC -3'; myc-BamHI Forward: 5'-CCATGGGATCCATGCAGAAGCTGATCTCAGAGG-3'; *ApaI Forward: 5'-GTT GTC TGC TGG GC**A** CCA TTC TTC CTC CAC-3'; and 3'-hMC4R SAC II Reverse: 5'-CCT CCC CGC GGA TAC CTG CTAGAC AAG TCA CAA AGG CCT CCC -3'. The first fragment was generated using the primers 3HA-BamHI Forward or myc-BamHI Forward and the reverse/antisense primer complementary to the forward sequence above (Apa I primer). The second fragment was generated using the 3'-hMC4R Sac II -Reverse primer and the forward/sense primer (ApaI sequence above). A 3xHA-hMC4R(R165W) cDNA (described above) served as template in a PCR reaction to generate a construct to be inserted in a targeting vector. Reaction conditions were 25 cycles of 94 °C (30 s), 67 °C (1 min), and 72 °C (1 min). Fragments were then

purified using the QIAGEN PCR purification kit (QIAGEN Mississauga, ON, Canada) and combined in an overlap extension reaction using 3HA-BamHI –Forward or myc-BamHI and 3’-hMC4R SacII -Reverse primers. Full-length PCR products were purified with QIAGEN gel extraction kit (QIAGEN Mississauga, ON, Canada) and digested with BamHI and SacII. All PCR products were sequenced to confirm the presence of desired modifications and absence of unwanted mutations.

Construction of the targeting vector

The right arm (RA) (DNA sequence from ENSMUSG00000047259) was amplified from genomic DNA extracted from an ES G4-129S6B6F1 cell line by PCR using primer 5’-RAForward (5’-CTA GCG GAT CCC GGG TGG GGG ACA GAG TGC AAA CTA GGT AGA TAC -3’) and primer 3’-RA Reverse (5’-ATT TGG AGC TCG TCG ACC TCA GTG TGT CTC AGG CTT G -3’). The resulting fragment was purified as described above, sequenced and digested with SacI and BamHI restriction endonucleases and ligated into a pBS-Bluescript SacI /BamHI vector. The long arm (LA) (DNA sequence from ENSMUSG00000047259) was amplified from genomic DNA extracted from an ES G4-129S6B6F1 cell line by PCR using primer LA#3 Forward (5’-GGG TAC CGT CGA CAA GCG AGG GAA CAG GGT CTC CAT AGA GAC-3’) and primer 3’-LA Reverse (5’-GGA GTG GAT CCT TCC TGC AGC AGC TGG ATT TGA GTC CTC C-3’) and the resulting fragment was purified as described above, sequenced and digest with KpnI and BamHI restriction endonucleases and ligated into a pBS-Bluescript -RA KpnI/BamHI vector. In order to flank the Neomycin selection cassette by FRT sites, PCR was performed from a pHR56 Neo plasmid vector (described in (2)) using 5’-NeoFRT Forward primer (5’-ATA TCA AGC TTG AAG TTC CTA TAC TTT CTA GAG AAT AGG AAC TTC TAC CGG GTA GGG GAG GCG

CTT TTC CCA AGG-3’) and 3’-NeoFRT Reverse primer (5’-AGC TGC CCG GGA AGT TCC TAT TCT CTA GAA AGT ATA GGA ACT TCA GCT TCT GAT GGA ATT AGA ACT TGG CAA AAC -3’). The resulting fragment was purified as described above, sequenced and digested with HindIII and SmaI restriction endonucleases, and ligated into a pBK-CMV HindIII /SmaI vector. In order to flank the Venus coding sequence by LoxP sites, PCR was performed from a pcDNA3.1-Venus Zeo (+) vector (kindly provided by Dr. Miyawaki) using 5’- VenLOX Forward primer (5’-TCT TTG GAT CCG CGG ATA ACT TCG TAT AGC ATA CAT TAT ACG AAG TTA TCC ATG GTG AGC AAG GGC GAG GAG CTG TTC ACC G -3’) and 3’-VenLOX Reverse primer (5’-TCA AAA AGC TTA TAA CTT CGT ATA ATG TAT GCT ATA CGAAGT TAT CTA CTT GTA CAG CTC GTC CAT GCC GAG AGT G-3’). The resulting fragment was purified as described above, sequenced and digested with BamHI and HindIII restriction endonucleases and ligated into a pBK-CMV-Neo BamHI/HindIII vector. The fragment 3HA-hMC4R(R165W) or myc-hMC4R(WT) BamHI /SacII was then ligated to the plasmid pBK-CMV-Neo-Venus BamHI /SacII. The plasmid pBK-CMV-3HA-hMC4R(R165W)-Venus-Neo or myc-hMC4R(WT)-Venus-Neo was digested with BamHI and SmaI restriction endonucleases. The fragment 3HA-hMC4R(R165W)-Venus-Neo or myc-hMC4R(WT)-Venus-Neo BamHI/SmaI was ligated to the plasmid pBS-Bluescript- LA-RA, which was cleaved beforehand with BamHI and SmaI restriction endonucleases to obtain the targeting vector. The plasmid was used to transform E. coli and amplified. Plasmid purification was done using a QIAGEN maxiprep kit (QIAGEN Mississauga, ON, Canada). The plasmid was cleaved by Sal I to linearize the targeting vector, consisting of 8.6kb, before electroporation in ES G4-129S6B6F1 cell lines. All PCR products were sequenced to confirm the presence of desired modifications and absence of unwanted mutations.

Generation of hMC4R knock-in mice

Targeted clones were identified by Southern blot analysis using ApaI digestion of ES cell genomic DNA and a labeled PCR-amplified DNA fragment derived from a flanking region 3' of the targeting construct as a hybridization probe (probe C). Cells selected for homologous recombination at the mMC4R locus were then electroporated with a plasmid coding for the Flip recombinase to excise the neomycin cassette prior to transfer of ES positive cells to blastocysts. New ES clones were screened by Southern blot hybridization analysis of SacI-digested-genomic DNA with the flanking probe C. ES clones with the predicted pattern were injected into C57BL6 blastocysts and germline-transmitting chimeric animals were obtained and then mated with C57BL6 mice. The resulting heterozygous offspring were crossed to generate non-transgenic littermates, heterozygous, and homozygous hMC4R knock-in mice. All mice were thus on a mixed C57Bl6/J and 129Sv background. Offspring were genotyped using the same strategy as for selecting ES neo-excised clones by Southern blot analysis.

Southern blot Hybridization

Genomic DNA from an ES G4-129S6B6F1 cell line or tail biopsies was prepared using a tissue DNA extraction kit (eZNA, D3396-02, OMEGA bio-tek, Norcross, GA, USA). 20ug of genomic DNA was digested overnight with the indicated restriction endonucleases, and electrophoresed through a 0.8% agarose gel. The digested DNA was subsequently transferred to an Amersham Hybond N+ nylon membrane (GE Healthcare: # RPN203B) by a capillary transfer method and hybridized with a ^{32}P-radiolabeled probe of 500 bp. To prepare the probe, the flanking Probe C at the mMC4R locus was amplified from genomic DNA extracted from an ES G4-129S6B6F1 cell line by PCR (annealing temperature 60°C, 30 cycles) using primer C forward: 5'-GGG CAT CCA

TGT GCA AAT CCG TAT CAA AGT -3’and primer C reverse: 5’-GGG CCC AAG CAC AGA CCC ATG TAT AAT TC -3’. The resulting fragment was purified as described above. The probe was labeled with ^{32}P-dCTP using the DECAprime II Random Primed DNA Labeling Kit (Ambion, Inc., Austin, TX, USA) according to the manufacturer’s instructions. Hybridization was performed in Ultrahyb Hybridization buffer (Ambion, Inc., Austin, TX, USA) and 106 cpm/ml of denaturated probe overnight at 42°C. The membrane was washed by successive washes in 2x SSC / 0.1% SDS for 20 minutes at50°C, 2x SSC / 0.1% SDS for 20 minutes at 55°C and 0.2x SSC / 0.1% SDS for 20 minutes at 60°C, and exposed to X-ray film for 48hrs at -80°C.

Gs activation BRET-based assay

Gαs protein engagement was measured between the plasma membrane marker rGFP-CAAX (3) and human Gαs-67-RlucII (4) in the presence of human Gβ1, Gγ9 and the tested receptor (mMC4R or hMC4R) using an ebBRET-based effector membrane translocation assay (5).

HEK293 clonal cell line (HEK293SL cells), described in previously published works (6, 7) were maintained in Dulbecco’s Modified Eagle Medium (DMEM, Wisent) supplemented with 10% fetal bovine serum (FBS, Wisent) and 1% antibiotics (100 U/mL penicillin and 100 μg/mL streptomycin (PS); Wisent). Cells were grown at 37°C in 5% CO2 and 90% humidity.

For BRET experiments, cells (3 mL at 3.5 x 10^5 cells per mL) were transfected with 126 ng Gαs-RlucII, 800ng Gβ1, 800ng Gγ9, 800ng rGFP-CAAX, 50ng MC4R DNA and completed to 2.8 μg total DNA amount with salmon sperm DNA (Invitrogen). Transfections were performed using a polyethylenimine solution (PEI, 1 mg/mL; Polysciences) diluted in NaCl (150 mM, pH 7.0; 3:1 PEI/DNA ratio). Following addition of cells to the stabilized DNA/PEI transfection mix, cells were immediately seeded (3.5 x 104 cells/well) into 96-well white microplates (Greiner Bio-one) and

maintained in culture for the next 48 h in DMEM containing 2% FBS and 1% PS. The day of the BRET experiment, cells were incubated in HBSS for 1 h at room temperature (RT). Cells were then treated with increasing concentrations of ligand (added by injection with a HP300e printer) along with the luciferase substrate coelenterazine prolume purple (1 μM, NanoLight Technologies) for 10 min at RT. BRET signal was detected in a SPARK 10M multimode plate reader (Tecan). The BRET signal was determined by calculating the ratio of the light intensity emitted by the acceptor (515 nm) over the light intensity emitted by the donor (410 nm).

Plasma quantitation of UM0130866 in homozygous R165W-hMC4R KI Mice

All in vivo procedures were conducted at Université de Montréal (Québec, Canada) under protocols approved by Université de Montréal institutional Animal Care and Use committee following all relevant guidance and regulations set by the Canadian Council on Animal Care in science. One of three doses (15, 60 or 100 mg/kg) of UM0130866: SDD, formulated in 0.5% methylcellulose in water, was administered to 14-18-week-old homozygous R165W-hMC4R KI mice by oral gavage. Conventional liquid chromatography tandem mass spectrometry was used to achieve separation and detection of analytes in plasma.

Quantitative RT-PCR

Briefly, hypothalamic area from frozen brain of adult mice was dissected using the brain slicer matrix (Zivic Instruments). Tissue was then homogenized in Trizol by trituration with up and down strokes through successively a18G needle then 22G and 26G needles and 1 ml syringe to obtain a complete homogenization.

Total RNA was evaluated for integrity with a 2100 Bioanalyzer (Agilent Technologies) and reverse transcribed into cDNA with the Maxima First Strand cDNA synthesis kit with ds DNase (Thermo Fisher Scientific). Gene expression was determined using assays designed with the Universal Probe Library from Roche (www.universalprobelibrary.com). For each qPCR assay, a standard curve was performed to ensure that the efficiency of the assay was between 90% and 110%. The QuantStudio7 qPCR instrument (Thermo Fisher Scientific) was used to detect the amplification level. mRNA expression is represented with a Ct value.

Three qPCR assays were designed, one detecting human MC4R and mouse Mc4r, a second detecting only human MC4R and a third detecting only mouse Mc4r. The human and mouse common assay showed a very similar expression throughout the samples, regardless of the genotype of the mouse (NTG, HET or HOMO). The assay detecting only human MC4R was showing expression only in HET and HOMO mice het and homo, and no expression in NTG mice. The assay detecting only mouse Mc4r showed expression only in NTG and HET mice. Mouse GAPDH was tested as an endogenous control and showed very similar expression throughout the samples.

The oligoes used are as follow:

Gene Symbol	UPL Probe	Fwd	Rev	RefSeq detected	Efficiency %
MC4R<br>HS and mm	76	tcacccatgtactttttcatctg	gaaacgctcaccagcatatc	NM_005912,<br>NM_016977	101
MC4R HS	86	atccatttgcagcctgctt	gttatggtactggagagcatagaaga	NM_005912	93
Mc4r mm	98	ggcgaggcttcacattaaga	gcccccttcatgttggta	NM_016977	110
GAPDH	80	tgtccgtcgtggatctgac	cctgcttcaccaccttcttg	NM_008084.2	91

Affinity for Melanocortin Receptors.

The affinities (Ki values) of UM0130866 for recombinant human MC3R, MC4R, and MC5R or

endogenous mMC1R were determined at CEREP using traditional radioligand displacement assays at the tracer concentrations indicated in Supplemental Table 2.

Supplemental References

1. Ho SN, Hunt HD, Horton RM, Pullen JK, and Pease LR. Site-directed mutagenesis by overlap extension using the polymerase chain reaction. *Gene.* 1989;77(1):51-9.
2. Metzger D, Clifford J, Chiba H, and Chambon P. Conditional site-specific recombination in mammalian cells using a ligand-dependent chimeric Cre recombinase. *Proc Natl Acad Sci U S A.* 1995;92(15):6991-5.
3. Namkung Y, Le Gouill C, Lukashova V, Kobayashi H, Hogue M, Khoury E, Song M, Bouvier M, and Laporte SA. Monitoring G protein-coupled receptor and beta-arrestin trafficking in live cells using enhanced bystander BRET. *Nat Commun.* 2016;7(12178.
4. Carr R, 3rd, Du Y, Quoyer J, Panettieri RA, Jr., Janz JM, Bouvier M, Kobilka BK, and Benovic JL. Development and characterization of pepducins as Gs-biased allosteric agonists. *J Biol Chem.* 2014;289(52):35668-84.
5. Avet C, Mancini A, Breton B, Le Gouill C, Hauser AS, Normand C, Kobayashi H, Gross F, Hogue M, Lukasheva V, et al. Selectivity Landscape of 100 Therapeutically Relevant GPCR Profiled by an Effector Translocation-Based BRET Platform. Submitted bioRxiv https://doi.org/10.1101/2020.04.20.052027).
6. Luttrell LM, Wang J, Plouffe B, Smith JS, Yamani L, Kaur S, Jean-Charles PY, Gauthier C, Lee MH, Pani B, et al. Manifold roles of beta-arrestins in GPCR signaling elucidated with siRNA and CRISPR/Cas9. *Sci Signal.* 2018;11(549).
7. Namkung Y, LeGouill C, Kumar S, Cao Y, Teixeira LB, Lukasheva V, Giubilaro J, Simoes SC, Longpre JM, Devost D, et al. Functional selectivity profiling of the angiotensin II type 1 receptor using pathway-wide BRET signaling sensors. *Sci Signal.* 2018;11(559).

Supplemental Figures and Tables

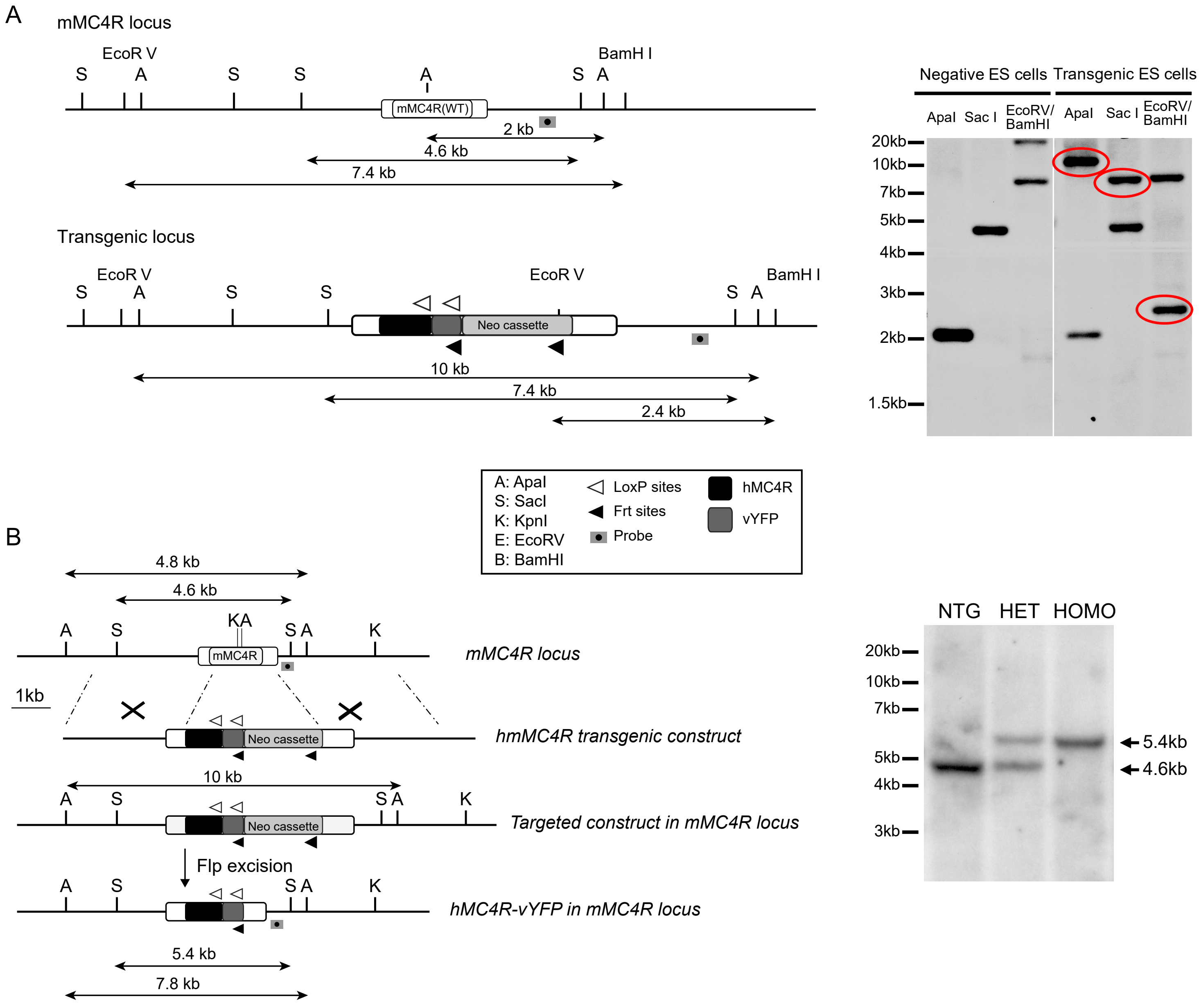

Supplemental Figure 1. Humanized KI mouse generation. (**A**) Left panel: Schematic representation of the homologous recombination at mMC4R locus in ES cells. The insertion of the human MC4R gene, the C-terminal YFP and the Neo cassette is shown. Right panel: Autoradiogram of a Southern blot analysis showing the different fragments of the mouse vs recombinant locus, with the new band at 10kb, 7.4kb and 2.4kb (circled in red) on top of the endogenous 2.4kb, 4.6kb and 7.4kb for SacI, ApaI and EcoRV/BamHI digests, respectively. (**B**) Left panel: schematic diagram of the KI strategy, replacing the *mc4r* gene by the targeting vector following homologous recombination. Right panel: Example of southern blot analysis of SacI-digested tail genomic DNA of R165W-hMC4R using the probe shown in the left panel.

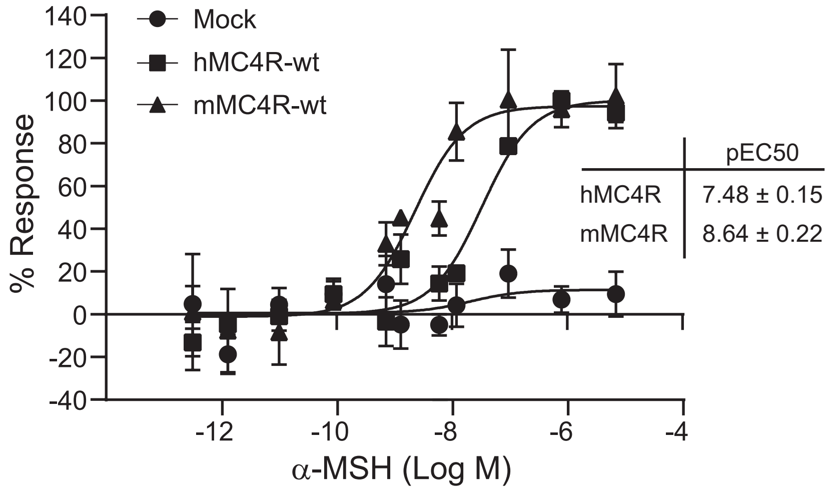

Supplemental Figure 2: Evaluation of the potencies of α-MSH to activate Gs protein following hMC4R or mMC4R stimulation. An ebBRET-based effector membrane translocation assay was used to monitor Gαs activation (5), measuring the Gαs dissociation from the plasma membrane upon receptor stimulation. Cells were transfected with hMC4R or mMC4R. Gs activation was measured by BRET between the Gs-RlucII (energy donor) and rGFP-CAAX (energy acceptor) following receptor stimulation with increasing concentration of α-MSH. Data are expressed as % response (maximal response = 100% for each receptor) and represent the mean ± SEM (mock n=6, hMC4R and hMC4R n=3). The potency values (pEC50) are presented and are the negative logarithm of the EC50 (ligand concentration giving 50% of the maximal response). To test whether LogEC50 for α-MSH differs between hMC4R and mMC4R, we performed the comparison of curve best-fit values using the Extra sum-of-squares F test from Prism version 8.4 (GraphPad Software, La Jolla California USA). LogEC50s were considered different with a P value equal to 0.0002.

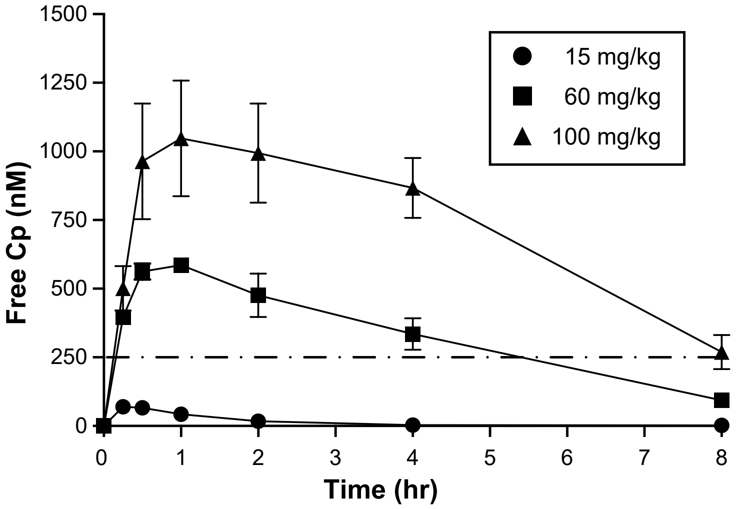

Supplemental Figure 3. Pharmacokinetic profile of the MC4R antagonist UM0130866. One of three doses [15 (circle), 60 (square) or 100 (triangle) mg/kg] of UM0130866: spray dried dispersion (SDD), formulated in 0.5% methylcellulose in water, was administered to 14-18-week-old homozygous R165W-hMC4R KI mice by oral gavage. UM0130866 plasmatic concentrations in blood were measured at the indicated time. Free plasmatic concentrations (Cp) were then calculated based on the protein binding capacity of the compound (mouse plasma binding: 96.6%). The dashed line represents the free concentration of the compound able to rescue 50% of the maximal efficacy of the R165W-hMC4R signaling (PC potency) (*in vitro* protein binding: 70%). Data reported are the means ±SEM of three animals per time point.

Supplemental Table 1: Quantitative RT-PCR of hypothalamic RNA extracted from homozygous and heterozygous hMC4R mice and compared to RNA extracted from non-transgenic littermate mice.

Species targeted	MC4R detection			GAPDH detection		MC4R /GAPDH		
	**Genotype**	**Ct Mean**	**Ct SD**	**Ct Mean**	**Ct SD**	**ratio**	**mean**	**SD**
**hMC4R**	HOMO	26.612<br>26.638<br>26.598	0.038<br>0.030<br>0.030	16.315<br>16.407<br>16.186	0.134<br>0.021<br>0.022	1.6311<br>1.6236<br>1.6433	1.63	0.01
**mMC4R**	NTG	25.571<br>25.320	0.105<br>0.053	16.428<br>16.201	0.259<br>0.298	1.5565<br>1.5629	1.56	0.00
**hMC4R and mMC4R**	HOMO	25.434<br>25.561<br>25.565	0.145<br>0.081<br>0.085	16.315<br>16.407<br>16.186	0.134<br>0.021<br>0.022	1.5589<br>1.5580<br>1.5794	1.57	0.01
	NTG	25.679<br>25.506	0.160<br>0.216	16.428<br>16.201	0.259<br>0.298	1.5631<br>1.5744	1.57	0.01
	HET	25.737<br>25.424	0.051<br>0.156	16.365<br>16.154	0.084<br>0.170	1.5727<br>1.5738	1.57	0.00

Data shown are the Ct values of each sample that represents mRNA expression level. Mouse GAPDH is used as endogenous control to correct for sample-to-sample variations.

Supplemental Table 2: Melanocortin receptor selectivity of UM0130866.

Ligand (UM0130866)	Ki (µM)	MC4R selectivity
**MC4R**	0.073	1
**MC1R**	17.9	245
**MC3R**	1.2	16
**MC5R**	0.56	8

The affinities (Ki values) of UM013866 for recombinant human MC3R, MC4R, and MC5R in CHO cells or for endogenous mMC1R in B16-F1 cells were determined using traditional radioligand displacement assays in the presence of ^{125}I-NDP-α-MSH (0.05 nM for MC1R, *K*d, 0.035 nM; 0.075 nM for MC3R, *K*d, 0.4nM; 0.05 nM for MC4R, *K*d, 0.54 nM; 0.05 nM for MC5R, *K*d, 0.7 nM).
